# Mesoscale cortical dynamics reflect the interaction of sensory evidence and temporal expectation during perceptual decision-making

**DOI:** 10.1016/j.neuron.2021.03.031

**Published:** 2021-06-02

**Authors:** Ivana Orsolic, Maxime Rio, Thomas D. Mrsic-Flogel, Petr Znamenskiy

**Affiliations:** 1 Sainsbury Wellcome Centre, University College London, 25 Howland Street, London W1T 4JG, UK; 2 Biozentrum, University of Basel, Klingelbergstrasse 70, 4056 Basel, Switzerland; 3 The Francis Crick Institute, 1 Midland Road, London NW1 1AT, UK; 4 The National Institute of Water and Atmospheric Research, 301 Evans Bay Parade, Hataitai, Wellington 6021, New Zealand

**Keywords:** decision-making, sensory processing, temporal expectation, wide-field calcium imaging, premotor cortex, secondary motor cortex, visual cortex, mouse behavior

## Abstract

How sensory evidence is transformed across multiple brain regions to influence behavior remains poorly understood. We trained mice in a visual change detection task designed to separate the covert antecedents of choices from activity associated with their execution. Wide-field calcium imaging across the dorsal cortex revealed fundamentally different dynamics of activity underlying these processes. Although signals related to execution of choice were widespread, fluctuations in sensory evidence in the absence of overt motor responses triggered a confined activity cascade, beginning with transient modulation of visual cortex and followed by sustained recruitment of the secondary and primary motor cortex. Activation of the motor cortex by sensory evidence was modulated by animals’ expectation of when the stimulus was likely to change. These results reveal distinct activation timescales of specific cortical areas by sensory evidence during decision-making and show that recruitment of the motor cortex depends on the interaction of sensory evidence and temporal expectation.

## Highlights

• Mice performed a visual change detection task based on uncertain sensory evidence
• Task design separated the processes of deliberation and action execution
• Sensory evidence modulated secondary motor cortex in absence of movement
• These responses were selective, sustained, and modulated by temporal expectation

## Teaser

Orsolic et al. reveal how the mouse neocortex is modulated by sensory evidence in a visual change detection task. They show that recruitment of the secondary motor cortex emerges with learning and reflects the interaction of sensory evidence and prior knowledge of task structure.

## Introduction

As animals form judgments about the sensory scene, information represented in sensory cortical areas influences motor actions by engaging a distributed network of sensorimotor pathways. Neural correlates of decision-making have been identified across modalities and species (Hanks and Summerfield, 2017) through recordings targeting individual brain areas (Newsome et al., 1989; Hanes and Schall, 1996; Shadlen and Newsome, 1996; Roitman and Shadlen, 2002; Romo et al., 2002; de Lafuente and Romo, 2005; Ding and Gold, 2010; Raposo, et al. 2014) or many brain areas in parallel (Hernández et al., 2010; Hanks et al., 2015; Siegel et al., 2015; Allen et al., 2017; Scott et al., 2017; Gilad et al., 2018; Zatka-Haas et al., 2018; Musall et al., 2019; Steinmetz et al., 2019).

Perceptual decisions involve interaction of sensory information with subjects’ expectations and prior knowledge leading up to behavioral choice (Gold and Shadlen, 2007; Summerfield and de Lange, 2014). Attributing neuronal responses to these pre-decision processes is challenging because they are inherently correlated with subsequent motor execution-related signals (Murakami and Mainen, 2015), which have a widespread effect on neural activity. Specifically, behavioral choice influences representation of sensory stimuli (Nienborg and Cumming, 2009), whereas neural correlates of task-related and spontaneous overt behaviors dominate global brain activity (Allen et al., 2017; Musall et al., 2019; Stringer et al., 2019). Although primate studies have set the gold standard in experimental design probing decision-making (Gold and Shadlen, 2007), the tools available with mice offer an opportunity to look at the distributed nature of decision-related processes. Mice can be trained in a range of perceptual tasks involving discriminating or detecting changes in visual stimuli (Harvey et al., 2012; Glickfeld et al., 2013; Poort et al., 2015; Burgess et al., 2017) and accumulating visual sensory evidence (Odoemene et al., 2018; Pinto et al., 2019).

To separate pre-decision processes from activity related to motor execution, we designed a behavioral task for mice that allowed us to independently probe the influence of sensory information and temporal expectation on neural activity while controlling the animals’ motor output. The task required mice to lick for reward in response to sustained changes in speed of a noisy drifting grating stimulus. Mice were encouraged to respond as soon as they detected the change by restricting the window when the reward was available. Because speed changes were often ambiguous, their timing variable, and the trial difficulty randomized, mice had to continuously monitor the sensory stimulus during an extended period preceding the change. Using a combination of experimental manipulations and post hoc analyses, we separated neural responses underlying evaluation of sensory evidence from those related to execution of motor responses. Importantly, by manipulating animals’ expectation of when sustained changes in speed were likely to occur, we determined how the responses to the same stimulus speed fluctuations were influenced by expectation of when the stimulus was likely to change.

We first identified the behavioral strategy used by the mice to detect sustained changes in stimulus speed, showing that they combine stimulus information on a timescale of hundreds of milliseconds with their prior expectation of the timing of changes. Using wide-field calcium imaging of the dorsal neocortex, we identified a cascade of activity induced by fluctuations in stimulus speed. Such fluctuations triggered transient responses in visual areas and culminated in more persistent activation of motor areas in the absence of choice execution. This recruitment of the motor cortex depended on the animals’ experience of the task and was modulated specifically by their temporal expectation of stimulus change. This localized pre-decision cascade contrasted with the widespread emergence of action-related signals associated with the execution of behavioral choice.

## Results

### Visual change detection task

**Figure 1:**
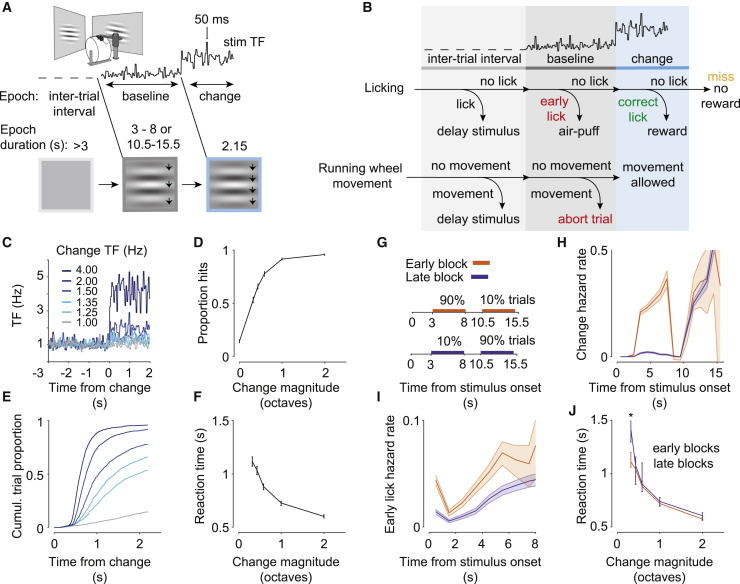
Stimulus speed change detection task and animal performance (A) Mice were head fixed, and two monitors were placed on each side of the animal. Trials consisted of three epochs: an inter-trial period with a gray isoluminant screen (light gray), a baseline stimulus period with a square patch of a drifting sine grating (dark gray, mean log_2_ TF of the grating = 0), and a change period when the speed of the stimulus increased (blue). Stimulus speed was updated every 50 ms so that the new log_2_ TF of the grating was sampled from a normal distribution (SD = 0.25 octaves), resulting in noisy drift of the grating. (B) Mice had to withhold licking and running wheel movement to initiate the stimulus presentation and throughout the baseline period until the change. If the mouse detected the change (correct lick), then a drop of soy milk was given as a reward. If the mouse licked before the change in the baseline period (early lick), an air puff was delivered. If the mouse missed the change, no air puff or reward was given. (C) Mice were trained to detect a range of stimulus speed increases (darker colors correspond to larger change magnitudes). (D) Mouse performance in detecting changes was modulated by change magnitude (6 mice, error bars indicate 95% CI, change magnitude is expressed in octaves as mean log_2_ TF). (E) Cumulative distributions of reaction times across stimulus speed changes. (F) Median reaction times are modulated by change magnitude (6 mice, error bars indicate 95% CI). (G) Timing of stimulus speed changes across early and late change blocks. (H) Probability of stimulus speed changes as a function of time (change hazard rate) in early and late blocks. Shading indicates 95% CI. (I) The probability of early licks as a function of time (early lick hazard rate) is modulated by anticipation of change. Shading indicates 95% CI. (J) At small change magnitudes, responses to early changes (3–8 s after stimulus onset) are slower in late blocks, when changes are not expected (p < 0.01, Wilcoxon rank-sum test). Error bars indicate 95% CI.

We trained head-fixed, food-restricted mice in a visual change detection task, which required them to lick for reward in response to a sustained increase (hereafter referred to as change) in the speed of a drifting grating stimulus (Figures 1A and 1B). The temporal frequency (TF) of the grating stimulus varied around the mean every 50 ms during the baseline (log_2_ TF = 0 ± 0.25) and the change periods on 70% of trials (referred to as noisy trials; Figure 1C). Such noisy trials provided a window to determine the strategies mice might use to perform the task and to probe stimulus-evoked modulation of cortical activity during decision-making (Huk and Shadlen, 2005; Hanks et al., 2015). After a randomly chosen delay period, the mean TF increased, and the mice were required to lick within a response window of 2 s to receive a drop of soy milk (Figure 1B). The frequency of correct licks depended on the magnitude of the TF change, with mice reliably detecting large increases in TF (Figure 1D; 19,734 noisy trials, 109 sessions, 6 mice). Reaction times were also modulated by the magnitude of stimulus changes, with mice responding more swiftly to larger increases in TF (Figures 1E and 1F). When mice licked prior to the change, the trial was aborted, and mice were penalized with an air puff to the cheek. In addition, we monitored their running speed and aborted the trial in response to movement of the running wheel during the baseline period (STAR Methods). We also collected a separate dataset from the same mice when they were free to run during the entire stimulus presentation (Figures S1A–S1C; 82,005 trials, 281 sessions, 6 mice).

To explore whether the timing of early licks was influenced by animals’ expectation of when changes might occur in the stationary version of the task, we varied the distribution of change times during the trial in blocks (Figures 1G and 1H). In early change blocks, changes occurred between 3 and 8 s after stimulus onset in most trials (90%) and between 10.5 and 15.5 s in the remaining trials (10%). The timings were reversed in late change blocks (Videos S1 and S2 show example early and late block trials). Mice varied the timing of early licks based on the probability of changes as a function of time (Figure 1H; probability of changes given that no change has occurred and the trial has not been aborted). The early lick hazard rate (the probability of licks occurring at a given time point given that no early licks or changes have yet occurred) was elevated at the start of the trial in early blocks (Figure 1I) when changes were more frequent. Mice were also faster to respond to the most difficult change during this period when the change was expected (Figure 1J). Therefore, prior expectation of when changes might occur influenced animals’ decisions to lick.

Video S1. Example correct detection trial in the early block, related to Figure 1 Playback at 0.5× speed.

Video S2. Example correct detection trial in the late block, related to Figure 1 Playback at 0.5× speed.

### A Gaussian process classification model uncovers stimulus features driving mouse behavior

**Figure 2:**
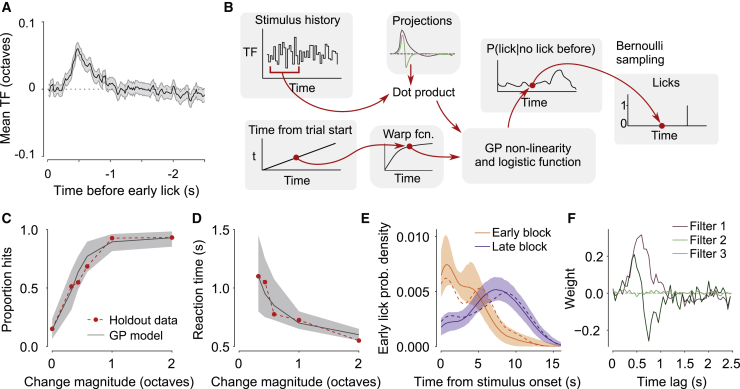
A GP classification model reveals animals’ behavioral strategy (A) Average stimulus preceding licks during the baseline stimulus (n = 6 mice, shading indicates 95% CI). (B) Structure of the GP classification model. (C–E) Model performance for an example mouse (425 holdout trials). The model captures the animal’s detection performance (C) and reaction times (D) as well as the timing of early licks (E), computed using a Gaussian kernel density estimate. Holdout data, dashed lines; model predictions, solid line and shading. Early and late block trials are combined in (C) and (D). To fit and evaluate the model, 14,944 behavioral trials were assigned to training, validation, and test subsets (8,966, 2,989, and 2,989 trials, respectively). Of these, 1,277, 424, and 425 trials were recorded during hazard rate manipulation sessions in stationary mice. During the remaining trials, mice were free to run during stimulus presentation. Shading indicates 2.5% and 97.5% quantiles of model predictions. (F) Principal filters learned during model optimization. The first two filters reveal the main stimulus features sufficient to capture mouse behavior.

Several behavioral strategies could explain the features of mouse behavior described above. For example, mice might decide to lick by integrating visual signals or by detecting fast outliers in the noisily drifting stimulus. Distinguishing between these strategies based on trial-average statistics is challenging (Brunton et al., 2013). To understand how sensory evidence is transformed into a decision to lick, we took advantage of the stochastic fluctuations in TF in noisy trials and examined the TF content of baseline stimuli preceding early licks by computing the lick-triggered average stimulus. Early licks were preceded by increases in temporal frequency spanning the period of ∼0.25–1 s prior to lick onset (Figure 2A). This observation shows that sensory information over this epoch contributes to animals’ behavior but does not unambiguously reveal how subjects weigh evidence in their decisions to lick (Okazawa et al., 2018).

To answer this question, we developed a statistical model optimized to predict the momentary lick hazard rate during sustained changes in stimulus speed and during the baseline period, based on the history of visual input (2.5 s) and time elapsed since the start of the trial (Figure 2B). To identify features of the stimulus that drove animals’ choices, we restricted stimulus information available to the model to a low-dimensional linear projection of stimulus history, defined as the convolution of stimulus history with a set of filter vectors. Filter outputs and elapsed time were combined by a Gaussian process (GP) non-linearity to estimate the log-odds of licking during each sample of the trial. The model assumes that the contributions of time and stimulus information to the log-odds of licking are additive, which is equivalent to combining current sensory evidence with prior odds of licking based on time since the start of the trial. Although this model has no direct biological interpretation, it provides an unbiased description of how mice transform stimulus information and time since trial start into licks, akin to linear-nonlinear-Poisson models used to characterize neuronal receptive fields. The model accurately captured the trial-average statistics of mouse behavior, including psychometric and chronometric curves (Figures 2C, 2D, and S2) and the timing of early licks (Figure 2E). The full model outperformed models that received stimulus or timing inputs alone (Figures S3A–S3H).

We next examined stimulus filters whose weights and numbers were optimized during model training. The shapes of the top two filters were consistent across all mice (n = 6) whose behavior we quantified (Figures 2F and S2). The weights of both filters were close to zero for time lags of 0–0.25 s, a period equivalent to non-decision time, reflecting sensory and motor delays. The first filter resembled the lick-triggered average stimulus and had large positive weights at time lags of ∼0.25–1 s and small negative weights at lags of 1–2 s (Figures 2F and S2). It is therefore sensitive to sustained increases in the TF of the grating over baseline. The second filter was almost symmetric and resembled a derivative filter, with positive weights between ∼0.25 and ∼0.5 s and negative weights between ∼0.5 and ∼1 s (Figures 2F and S2D). It is therefore sensitive to abrupt changes in TF of the grating. The weights of the remaining filters were close to zero, with the exception of the third filter in 2 of 6 mice, which resembled the derivative filter but was shifted in time (Figure S2D).

To characterize the contribution of the two filters to model performance, we examined GP models with coefficients of either of the two filters set to 0. Eliminating the first filter dramatically reduced the proportion of hit licks across all stimulus change magnitudes in stationary and running mice (Figures S3I and S3L). On the other hand, eliminating the second filter had a more subtle effect on performance, primarily affecting animals’ responses in the running version of the task (Figure S3I) and increasing reaction times, especially for large stimulus strengths (Figures S3J and S3M). Both filters were more active at the time of hits rather than early licks, with the first filter showing graded activation across all stimulus change magnitudes and the second filter being consistently active for large changes only (Figures S3K and S3N). Thus, both filters primarily contribute to licks during the change period, with the first filter playing a more prominent role. These analyses suggest that mouse behavior is primarily explained by a strategy involving integration of TF on the timescale of ∼1 s and by the expectation of when the sustained changes in stimulus speed might occur.

### Imaging neural activity across the dorsal cortex during the task

**Figure 3:**
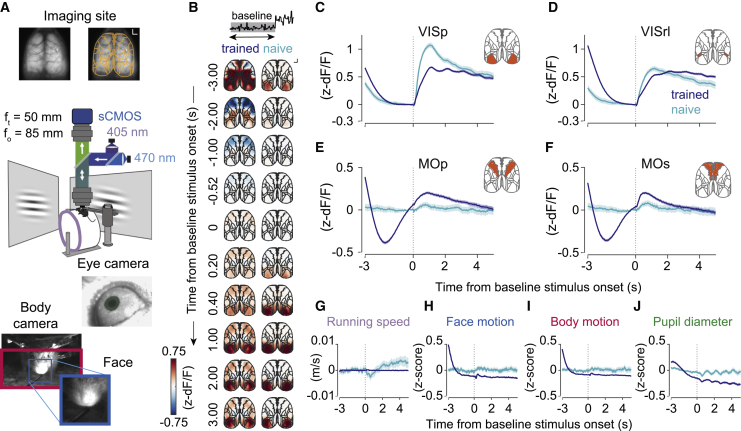
Stimulus onset broadly activates the dorsal cortex in trained animals (A) Top: extent of the imaging site; orange, outlines of regions of interest (ROIs) analyzed after brain registration. Outer borders were cropped to the extent of the imaging site. Center: schematic of the behavioral setup and wide-field macroscope. Bottom: example images from cameras capturing the animals’ pupil (green), face (cyan), and body movements (magenta). (B) Mean *Z*-scored fluorescence response around the onset of the baseline stimulus in trained (left, 6 mice) and naive animals (right, 3 mice). Inset: shading indicates the analyzed trial epoch. Scale bar, 1 mm. (C–F) Mean *Z*-scored responses of selected cortical areas around the onset of the baseline stimulus in trained and naive animals. Vertical lines mark baseline stimulus onset. Prior to stimulus onset, activity in the primary visual area (VISp) and rostrolateral visual area (VISrl) in trained and naive animals decreased, likely reflecting the offset of the visual stimulus in the previous trial. In trained animals only, this decrease in activity was also present in the primary motor area (MOp) and secondary motor area (MOs), coincident with a reduction in overt movements (H–I), followed by an anticipatory increase in activity prior to stimulus onset (shading indicates 95% CI). (G–J) Quantification of overt movements in trained and naive animals in response to stimulus onset (traces are corrected by mean value in a 0.5-s window before the stimulus; baseline values at time 0 are stated in brackets, shading indicates 95% CI) of (G) running speed (trained, 8.35 × 10^−5^; naive, 0.02 m/s), (H) face motion (trained, −0.49; naive, −0.06), (I) body motion (trained, −0.56; naive, −0.03), and (J) pupil diameter (trained, −0.11; naive, 0.01).

We next systematically characterized the patterns of neural activity underlying processing of sensory signals and their transformation into putative motor commands across the dorsal cortex. To accomplish this, we imaged transgenic mice expressing GCaMP6s in excitatory cortical neurons (Wekselblatt et al., 2016; 11,130 trials, 47 sessions, 6 mice) using a low-magnification epifluorescence microscope that allowed us to simultaneously capture bulk calcium signals across the entire dorsal surface of the mouse neocortex (Figure 3A; STAR Methods). To compensate for changes in fluorescence arising from hemodynamic fluctuations, we interleaved illumination at 470 nm and 405 nm and used frames acquired at 405 nm to estimate the hemodynamic component (Allen et al., 2017). In parallel, we monitored animals’ pupil diameter and body and face (snout region) movements (Figure 3A). To identify the imaged brain areas, at the end of each imaging experiment we reconstructed whole-brain volumes using serial two-photon tomography and defined cortical area boundaries based on the Allen Mouse Brain Common Coordinate Framework (CCF; v.3; Figures 3A and S4; STAR Methods). In the presentation of our results, we focus on responses in four cortical areas, which show markedly different patterns of wide-field activity during baseline and change periods: the primary and rostrolateral visual areas (VISp and VISrl, respectively) and primary and secondary motor areas (MOp and MOs, respectively). Responses in all imaged regions of interest (ROIs) are presented in the Supplemental information.

### Visual stimulus onset engages a distributed cortical network in trained mice

We first analyzed dorsal cortical activation patterns around the time of onset of the baseline stimulus (Figure 3B). In trained animals, trial onset was preceded by an anticipatory increase in MOp and MOs activity (Figures 3E and 3F). Presentation of the baseline stimulus triggered sustained activation of primary and secondary visual areas (Figures 3C and 3D; 6,631 noisy trials longer than 1.5 s, 47 sessions, 6 mice; Video S3), followed by recruitment of the secondary motor area (Figure 3F). Although grating onset triggered responses of similar or even larger magnitude in visual areas in naive mice, onset responses in the secondary motor area were markedly weaker (1,680 noisy trials from 10 sessions in 3 mice; Video S3). Thus, we observed strong recruitment of the secondary motor cortex by onset of the visual stimulus even in the absence of movement (Figures 3G–3J) that depended on animals’ experience of the task.

Video S3. Maps of mean *Z*-scored fluorescence responses aligned to onset of the baseline stimulus in trained (left) and naive (right) animals, related to Figure 3 Scalebar, 1 mm. Playing 0.5× speed.

### Action-related signals are represented throughout the dorsal cortex

**Figure 4:**
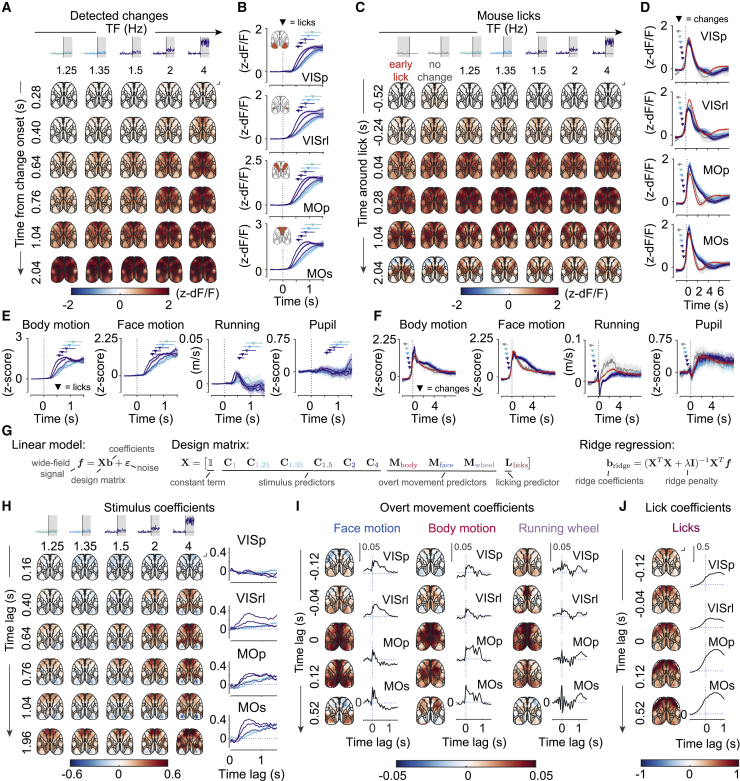
Wide-field calcium responses across the dorsal cortex during the change period are global and dominated by action-related activity (A and C) Maps of mean *Z*-scored fluorescence across the dorsal cortex aligned to change onsets on hit trials (A) or lick onsets (C), sorted by change strength. Insets: shading indicates the analyzed the trial epoch, and color indicates change strength. Scale bars, 1 mm. (B and D) Mean *Z*-scored fluorescence of selected cortical areas aligned to change onset in hit trials (B) and lick onsets (D). Shading indicates 95% CI. (E and F) Quantification of overt movements aligned to change onset in hit trials (E) and lick onset (F). Horizontal lines and markers represent the interquartile range and median reaction times (E) or stimulus change times (F). Shading indicates 95% CI. (G) Summary of the ridge regression model used to separate the contributions of task events and overt movements to wide-field fluorescence. (H) Model coefficients corresponding to change onsets across change magnitudes and time lags corrected by coefficients for 1-Hz (no change) trials across the dorsal cortex and for cortical areas shown in (B). Scale bar, 1 mm. (I and J) Model coefficients corresponding to overt movements and licks across time lags across the dorsal cortex and for cortical areas shown in (B). Scale bar, 1 mm.

We next examined the patterns of activity evoked by sustained changes in TF of the grating the mice were trained to detect. Change onset triggered an increase in wide-field fluorescence across the dorsal surface of the cortex in hit trials (Figure 4A; 1,974 noisy trials; Video S4). These responses were apparent earliest in motor areas, reaching half-max (50% of maximum response) 0.48 s after change onset for the strongest stimuli (MOs) compared with 0.6 s for the primary visual area (VISp) (Figures 4B and S5A). In more difficult trials, half-max latencies followed the increase in reaction times. Across stimulus strengths, the time course of neural responses followed the movement of the mouse, as captured by the body camera even prior to detection of licks (Figures 4E, S5B, and S5C). This widespread modulation of cortical activity was not observed in miss trials (Figures S5D and S5E; 463 noisy trials).

Video S4. Maps of mean *Z*-scored fluorescence responses aligned to change onset in hit trials, sorted by stimulus strength (left to right: 1.25, 1.35, 1.5, 2, and 4 Hz), related to Figure 4 Scalebar, 1 mm. Playback at 0.5× speed.

When aligned to the onset of licking, wide-field fluorescence responses were stereotyped across stimulus change strengths (Figures 4C, 4D, S6B, and S6C; Video S5) and were similar to early lick responses (Figures 4C, S6D, and S6E; 1,564 noisy trials). Although lick-related activity was global, it did not appear synchronously across the cortex. It was detectable earliest in the secondary motor area and anterior visual and midline areas (anterolateral visual area [VISal], 360 ms prior to lick; anteromedial [VISam], anterior [VISa], rostrolateral visual area [VISrl], MOs, retrosplenial [RSPd], and anterior cingulate area [ACAd], 320 ms; quantified as the time to cross 10% of maximum response on 1.5-Hz change trials). The difference in timing of lick-aligned responses in the VISp and MOs was apparent across change strengths (Figure S6A).

Video S5. Maps of mean *Z*-scored fluorescence responses aligned to hit licks, sorted by stimulus strength (left to right: 1.25, 1.35, 1.5, 2, and 4 Hz), related to Figure 4 Scalebar, 1 mm. Playback at 0.5× speed.

Lick-aligned activity dominated but did not fully account for cortical responses following onset of sustained changes in stimulus speed. To illustrate this, we first examined activity in hit trials with long reaction times (>0.84 s). During these trials, change onset triggered a gradual increase in fluorescence, which was modulated by the strength of the stimulus (Figure S5F). However, although no licks were present during this period, this activity was correlated with other overt movements preceding licking, as captured by the body camera (Figure S5G).

We next used ridge regression to fit a linear model using stimulus changes and overt body movements as predictors of wide-field fluorescence (Figure 4G). Accounting for execution of motor responses revealed components of wide-field fluorescence related to processing of the sensory stimulus distinct from the global responses following the change (Figure 4H). Based on this regression analysis, change onset was associated with activation of anterior higher visual areas (e.g., area VISrl) and of the secondary motor cortex and with a modest reduction in wide-field fluorescence of the primary visual cortex. The signs of change responses in areas VISp and VISrl may reflect the typical temporal frequency preferences of neurons in these areas (Andermann et al., 2011; Marshel et al., 2011), whereas the relatively small magnitude of VISp responses may be a consequence of bulk averaging of neurons with heterogeneous selectivity. Responses of higher visual areas and the secondary motor cortex were modulated by the strength of the stimulus (Figure 4H). Licking was associated with a widespread increase in activity, with strongest modulation of anterior regions of the primary and secondary motor cortices (Figure 4J), consistent with previous reports of involvement of these areas in licking behavior (Guo et al., 2014).

These analyses reveal distinct patterns of activity following change onset related to processing of the stimulus and execution of licking responses. The secondary motor cortex is engaged by both of these processes, implicating it in transformation of sensory evidence leading up to animals’ choices.

### Fluctuations in sensory evidence prior to choice trigger a localized cascade of wide-field activity ranging from transient responses in visual areas to sustained responses in motor areas

**Figure 5:**
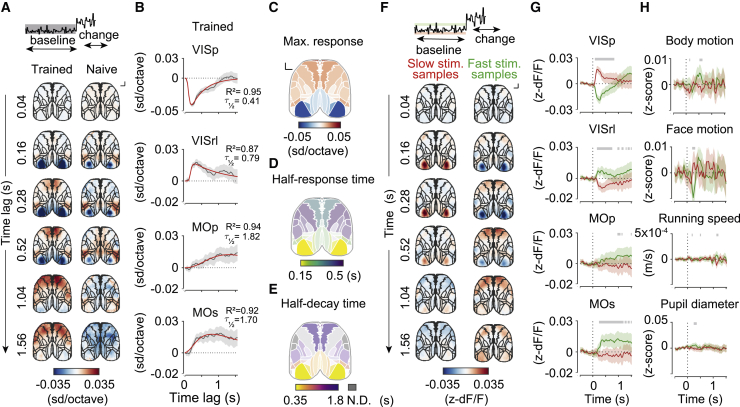
Baseline stimulus fluctuations trigger a localized cascade of activity across the dorsal cortex in the absence of overt movements (A) Maps of regression coefficients of wide-field fluorescence against baseline stimulus TF in trained (left) and naive (right) animals across time lags. The units are SD (changes in *Z*-scored wide-field fluorescence) per octave (log_2_-transformed stimulus TF). The color indicates the sign (red, positive; blue, negative) and saturation the strength of the relationship between fluorescence and baseline stimulus fluctuations. Scale bar, 1 mm. (B) Time course of regression coefficients of wide-field fluorescence against baseline stimulus fluctuations in example cortical regions; regression coefficients (gray, 95% CI) and multiexponential fit (red). Time lags when the CI does not include zero indicate significant responses. (C) Magnitude of modulation of cortical areas by subthreshold stimulus fluctuations. (D) Response latency (time to half maximum) across cortical areas. Saturation is scaled based on response magnitude (C). (E) Response half-decay time across cortical areas. Saturation is scaled based on response magnitude (C). Regions for which half-decay time could not be determined are shaded gray. (F) Maps of mean *Z*-scored fluorescence responses to slow (anti-licking, red) and fast (pro-licking, green) subthreshold stimulus fluctuations in trained mice (6 mice). Scale bar, 1 mm. (G and H) Mean *Z*-scored fluorescence of selected cortical areas (G) and quantification of overt movements (H) aligned to slow (red) and fast (green) baseline stimulus fluctuations in trained mice. Shading indicates 95% CI. Gray bars indicate significant differences between responses to fast and slow fluctuations (two-sample t test, p < 0.05).

We next focused on the extended baseline period of the task when mice observed subthreshold stimulus fluctuations and refrained from overt movement. This allowed us to determine the influence of sensory evidence on wide-field cortical activity and its time course and interaction with temporal expectation while directly controlling for the motor confounds described above. To characterize the temporal progression of visual stimulus processing in the dorsal cortex, we quantified the effect of sensory evidence during the baseline stimulus on wide-field fluorescence at different time lags using linear regression (Figures 5A, 5B, S7A, and S8A; Video S6; 1,039,391 stimulus samples from 6,894 trials). To ensure that movement-related activity immediately preceding licks did not affect this analysis, we excluded fluorescence frames from trials interrupted because of early licks or movement acquired less than 1 s prior to these events as well as frames following change onset.

Video S6. Maps of regression coefficients of wide-field fluorescence against baseline stimulus temporal frequency in trained (left) and naive (right) animals, related to Figure 5 Scalebar, 1 mm. Playback at 0.25× speed.

The temporal frequency of the baseline stimulus was correlated negatively with bulk activity of the primary visual cortex but correlated positively with that of anterior higher visual areas (Figures 5A–5C). The latencies (defined as time until 50% of maximum response; Figure 5D) of these responses were shortest in the VISp (0.15 s) and posteromedial visual area (VISpm; 0.16 s), followed by anterior higher visual areas: VISrl (0.21 s), VISa (0.22 s), and VISal (0.25 s). Because wide-field calcium signals represent bulk averages of largely local population activity (Ma et al., 2016; Makino et al., 2017), this modulation is consistent with the typical preference of the primary visual cortex and higher visual area neurons. Although VISp neurons tend to prefer slow visual speeds, neurons in the VISal, VISrl, and VISa respond preferentially to high speeds (Andermann et al., 2011 Marshel et al., 2011). Similar responses in visual areas were also present in naive mice, consistent with their sensory-driven origins (Figure 5A). In trained mice, modulation of visual areas by temporal frequency was followed by activation of the secondary motor area (MOs; 0.33 s) and weaker recruitment of the primary motor cortex (MOp; 0.63 s). Unlike responses in visual cortical areas, modulation of motor cortical activity was not observed in naive mice (Figure 5A; 362,291 frames from 1,680 trials; Video S6), indicating that recruitment of these areas was dependent on learning.

Cortical areas also differed in the offset dynamics of the wide-field responses. Bulk activity in the primary visual cortex decayed rapidly to baseline (VISp, half-decay time of 0.41 s; Figures 5B and 5E), suggesting that it is largely modulated by the immediate history of sensory stimulation. These responses in the primary visual cortex were similar to the reported half-decay time for somatic signals in GCaMP6s transgenics (Dana et al., 2014) and provide an estimate of the indicator offset kinetics of wide-field signals in this study. In contrast, responses in higher visual areas and motor areas were sustained (VISrl, 0.79 s; VISal, 0.93 s; VISa, 0.97 s), with half-decay times exceeding 1.5 s in the MOs (1.7 s) and MOp (1.82 s) (Figures 5B, 5E, and S7A).

The regression analysis described above revealed the sign and time course of modulation of the wide-field activity of the dorsal cortex by the visual stimulus. To characterize this relationship in more detail, we computed mean responses to the extremes of the stimulus during the baseline period, which carry different information for the animal: fast (pro-licking, n = 41,194) and slow (anti-licking, n = 42,253) stimulus samples (1.5 standard deviations above or below the mean TF, respectively), using responses to stimulus near the mean TF (±0.5 standard deviations, n = 467,681 samples) as a reference (Figures 5F, 5G, and S8B; Video S7; all imaged cortical areas shown in Figure S7B). In the primary visual cortex and VISpm, fast stimulus samples were associated with a decrease in bulk fluorescence compared with the reference stimulus response, whereas slow stimulus samples triggered an increase in bulk fluorescence (Figures 5F, 5G, and S7B). These effects were reversed in the VISrl, VISal, and VISa (Figures 5F and S7B). In contrast, motor areas were activated preferentially by fast stimulus samples, whereas slow samples had no significant effect (Figures 5F and 5G). Thus, the secondary and primary motor cortex were recruited specifically by stimulus fluctuations mice were trained to detect.

Video S7. Maps of mean *Z*-scored fluorescence responses to pro-licking (fast, left) and anti-licking (slow, right) subthreshold stimulus fluctuations in trained mice (6 mice), Related to Figure 5 Scalebar, 1 mm. Playback at 0.25× speed.

In the above analyses, we minimized the effect of movement-related activity on our estimates of neural responses by limiting our analysis to periods when the mice refrained from licking and moving the wheel. However, other movements, such as whisking or postural adjustments, could still occur sporadically without interrupting the trial and could contribute to the observed wide-field responses. To control for this possibility, we quantified animals’ movements in response to fast and slow stimulus samples as captured by the body camera. Fast stimulus samples triggered a small but significant decrease in face movement followed by a small increase in body motion (Figure 5H). To account for these differences in behavior, we used ridge regression to fit a linear model of wide-field fluorescence, including the baseline stimulus and videography data capturing overt movements as predictors (Figures S9A–S9C). This analysis confirmed that the pattern of wide-field activity triggered by baseline stimulus fluctuations could not be explained by these small overt movements. Additionally, we used ridge regression to fit a linear model of wide-field fluorescence that only included overt movements as predictors (Figures S9D and S9E). We then repeated the same analysis as described earlier (Figures 5A and 5B) on residuals of this model. This analysis again confirmed that distinct time courses of cortical engagement by sensory evidence, culminating in recruitment of the motor cortex, cannot be trivially accounted for by overt movements.

In a different variant of the task, mice were required to run on the wheel to initiate a trial and were free to run during stimulus presentation. Although not instructed in the task, mice changed their running speed during the baseline grating stimulus. Specifically, their average running speed decreased over time during the baseline stimulus (Figure S1E), perhaps reflecting the temporal structure of the task. Additionally, mice sped up after slow stimulus samples and slowed down after fast stimulus samples (Figures S1H and S1I). The resulting correlation between baseline stimulus TF and running speed confounded the interpretation of wide-field fluorescence responses. In contrast to the localized cascade of activity we observed in the stationary version of the task, fast stimulus samples in running mice triggered widespread modulation of dorsal cortical activity (Figures S1G and S10; Video S8). The time course of these widespread responses resembled that of running behavior but was opposite in sign (Figure S10A). These observations highlight the importance of controlling for task-instructed and non-instructed movement in interpretation of neural data (Musall et al., 2019; Stringer et al., 2019) and motivated us to focus our analyses on the stationary version of the task.

Video S8. Maps of mean *Z*-scored fluorescence responses to pro-licking (fast) subthreshold stimulus fluctuations in trained mice (6 mice) in the stationary (left) and running (right) versions of the task, related to Figure 5 Scalebar, 1 mm. Playback at 0.25× speed.

### MOs wide-field responses to baseline stimulus fluctuations reflect local population activity

**Figure 6:**
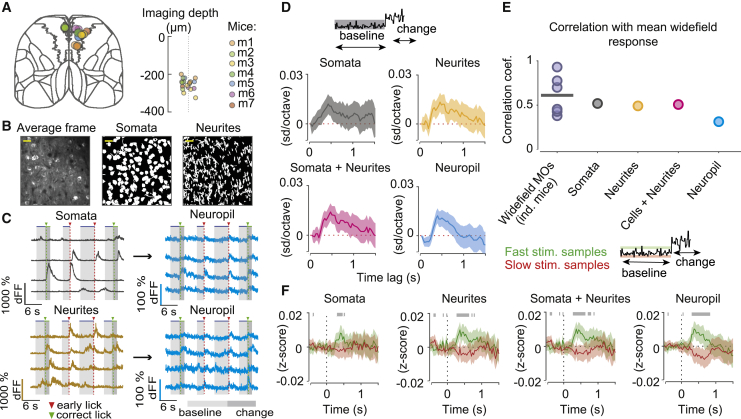
Average two-photon calcium responses to baseline stimulus fluctuations in the secondary motor cortex are consistent with wide-field signals (A) Localization of two-photon imaging sessions in the CCF (left) and depth from the cortical surface (right). (B) Two-photon imaging site, average frame (left) and imaging site segmentation into somata (center) and neurites (right). Scale bar, 25 μm. (C) Example single trial responses of individual two-photon ROI categories. Each row is an example trace from individual somata or neurites and the neuropil surrounding them (note the difference in scale). (D) Regression coefficients of average responses of ROI categories (*Z*-scored averages) and baseline stimulus fluctuations across different time lags (shading indicates 95% CI). (E) Comparison of the average wide-field MOs response and the responses of two-photon ROI categories (Pearson correlation of their average time courses in a 0- to 1.48-s window). As a measure of animal-to-animal variability, responses of individual mice in the wide-field cohort were compared with the average wide-field response in the remaining mice (line, mean across mice). (F) Mean *Z*-scored fluorescence responses to slow (anti-licking, red) and fast (pro-licking, green) baseline stimulus fluctuations across two-photon ROI categories. Shading indicates 95% CI. Gray bars indicate significant differences between responses to fast and slow fluctuations (two sample t test, p < 0.05).

Wide-field calcium signals are thought to reflect largely local population activity of superficial cortical layers (Ma et al., 2016; Allen et al., 2017; Makino et al., 2017), including neuropil, local somata, and dendrites from neurons in deeper layers. To confirm that the wide-field responses in the secondary motor cortex are consistent with the activity of the local population, we used two-photon calcium imaging in mice performing the task (Figures 6A and 6B). We examined the average activity of three ROI categories representing possible sources of the wide-field signal: somata, neurites (mostly dendrites), and adjacent neuropil (Figures 6B and 6C; STAR Methods). Somata as well as other ROI categories showed sustained modulation by stimulus fluctuations (Figure 6D; 5,795 trials, 974,470 stimulus samples, 19 sessions, 7 mice), demonstrating that MOs neurons are driven by sensory evidence during the baseline period, as observed in wide-field signals. Furthermore, the time course of wide-field signals was most similar to that of somatic responses (Figure 6E), comparable with the similarity of responses of individual wide-field animals to the rest of the wide-field cohort. Although neuropil fluorescence was also modulated by baseline stimulus fluctuations (Figure 6D), this modulation may reflect the contribution of dendrites and axons of local somata as well as long-range inputs. Analysis of responses to extreme stimulus fluctuations across different ROI categories found that, although MOs somata and neurites were modulated primarily by fast stimulus samples, in agreement with the wide-field responses, neuropil was modulated significantly by fast and slow stimulus samples (Figure 6F; fast pro-licking samples, n = 63,216; slow anti-licking samples, n = 65,201; relative to samples near the stimulus mean, n = 381,865).

These results suggest that the responses of layer 2/3 neurons in the secondary motor cortex to baseline stimulus fluctuations are consistent with the observed wide-field MOs responses but do not exclude that other sources, such as apical dendrites of deep-layer neurons, could also contribute to the observed wide-field signals (Peters et al., 2021).

### Temporal expectation modulates activation of the motor cortex by sensory evidence

**Figure 7:**
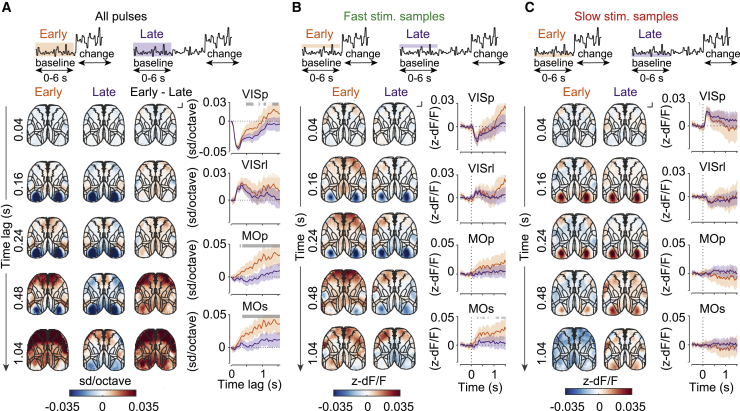
Modulation of wide-field responses to stimulus fluctuations by temporal expectation (A) Regression coefficients of wide-field fluorescence against baseline stimulus temporal frequency during 0–6 s of the trial in early (orange) and late (purple) change blocks and difference between the blocks (early-late). Shown are maps (left) and selected ROIs (right). Shading indicates 95% CI. Gray bars indicate significant differences between early and late blocks (STAR Methods). Scale bar, 1 mm. (B and C) Mean Z-scored fluorescence responses to fast (pro-licking, B) and slow (anti-licking, C) subthreshold stimulus fluctuations during 0–6 s of the trial in early and late change blocks. Notation as in (A). Gray bars indicate significant differences between early and late blocks (two-sample t test, p < 0.05)..

When making decisions, animals take advantage of immediate sensory evidence as well as their predictions of environmental events, but how the cortex combines these signals is unknown. To answer this question, we determined how the wide-field responses to fluctuations in sensory evidence during the baseline stimulus were influenced by animals’ temporal expectation of stimulus change. We compared the relationship between stimulus speed and wide-field fluorescence during the same trial period between trials when animals were expecting a change to occur (in early change blocks) and when the change was not expected (in late change blocks) using linear regression as above (Figure 7A; 244,491 stimulus samples from 2,521 trials in the early block and 521,827 stimulus samples from 4,373 trials in the late block). We focused on the period when the early lick hazard rate differed between blocks (0–6 s after baseline stimulus onset; Figure 1I).

We found that animals’ temporal expectation of stimulus speed change specifically modulated the relationship between stimulus speed and wide-field fluorescence in motor areas (Figures 7A and S11A). The secondary and primary motor cortices responded more strongly to stimulus fluctuations during early blocks, where speed changes were expected soon after the start of the trial. In contrast, initial responses to sensory evidence in visual areas were indistinguishable between early and late blocks (Figures 7A and S11A), with significant differences emerging only following peak response time.

To understand how animals’ temporal expectation affected the wide-field responses to fluctuations of sensory evidence in the motor cortex, we computed mean responses to fast (pro-licking) and slow (anti-licking) stimulus samples during the first 6 s of the trial in early and late change blocks (Figures 7B and 7C; early change block: fast stimulus samples, n = 9,655; slow stimulus samples, n = 9957; reference samples, n = 110,282; late change block: fast stimulus samples, n = 20,864; slow stimulus samples, n = 21,111; reference samples, n = 234,372). Responses to slow stimulus samples were similar between the two change blocks. On the other hand, responses to fast stimulus samples in the secondary motor cortex increased when animals were expecting the change. Overt mouse behavior in the same period showed small but significant differences between the two expectation blocks (Figures S11B and S11C). However, controlling for movement-related activity using ridge regression (Figure S11D) did not explain the modulation of wide-field fluorescence by expectation in motor areas (Figures S11E and S11F). Thus, temporal expectation influences engagement of the secondary and primary motor cortex by sensory evidence.

## Discussion

### Visual change detection as a paradigm to study perceptual decisions

To study the neural correlates of computations underlying perceptual decisions, we developed a visual change detection task where mice had to report sustained changes in speed of a noisy stimulus. We identified the strategy used by mice in the task using a combination of model-based and model-free approaches. This analysis suggested that mouse behavior was best explained by a combination of two stimulus filters: the primary filter, reflecting the average of the stimulus speed on a timescale of ∼1 s, and a secondary filter attuned to abrupt steps in speed. Finally, by manipulating the timing of changes during the trial, we showed that animals’ expectation of when stimulus speed changes might occur contributed to their decision to lick.

**Figure 8:**
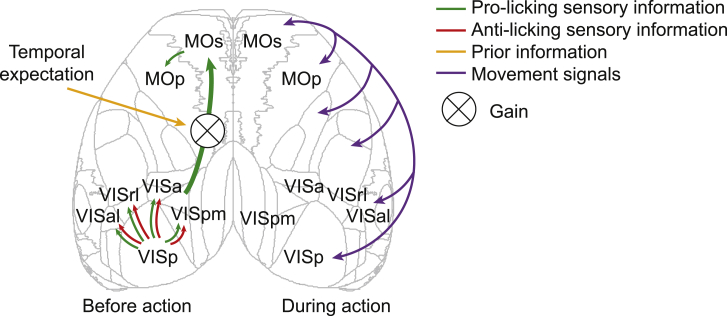
Proposed functional flow of sensory- and movement-related signals in the mouse dorsal cortex based on wide-field signals during the change detection task Sensory information modulates activity in primary and higher visual areas. Pro-licking sensory signals modulate wide-field activity in the secondary motor cortex on extended timescales. Animals’ expectation modulates the gain of secondary motor cortex responses to sensory evidence. When the animal executes its choice, movement-related signals are broadcast broadly across the dorsal cortex.

Typically, neural activity in reaction time tasks reflects the interaction of multiple concurrent and correlated signals, including those related to sensory integration, action selection, and execution (Park et al., 2014). The baseline period of our task allowed us to independently characterize the patterns of neural activity underlying processing of sensory evidence separate from the responses associated with the execution of behavioral choice (Figure 8). By taking advantage of the stochastic nature of stimulus speed during the prolonged baseline period, we used wide-field imaging across the dorsal neocortex to uncover the patterns of population activity preceding the commitment to a decision, reflecting the transformation of sensory evidence and its interaction with animals’ expectation while controlling for overt motor responses.

### A localized cascade of cortical activity reflects pre-decision processing of sensory evidence

We found that stimulus speed fluctuations during the baseline period specifically modulated wide-field activity of several areas of the dorsal cortex. This modulation differed in sign and temporal dynamics across areas. Tracking the ongoing stimulus, areas VISp and VISpm responded transiently and bidirectionally to pro-licking (high speed) and anti-licking (low speed) stimulus information with short latencies. In areas VISa, VISal, and VISrl, which form the core of the mouse posterior parietal cortex (Hovde et al., 2019), bidirectional responses to fluctuations in the visual stimulus speed were sustained over hundreds of milliseconds. This observation is consistent with electrophysiological recordings in rats that suggest that the posterior parietal cortex faithfully represents accumulated sensory evidence (Hanks et al., 2015). Wide-field activity of visual cortical areas contrasted with that of the secondary motor cortex, which responded selectively to transient pro-licking stimulus samples in a sustained manner, with fluorescence signals persisting on a timescale exceeding 1 s. The difference in persistence of stimulus responses between visual and motor areas is consistent with the hierarchy of activity timescales reported in primate and rodent studies (Murray et al., 2014; Pinto et al., 2020). Although sensory responses in primary and secondary visual cortical areas were present in trained and naive mice, modulation of motor cortex wide-field fluorescence by sensory stimulus fluctuations was observed only in trained animals and was not accounted for by overt movements. These observations suggest that acquisition of the task shaped the flow of sensory information in the dorsal cortex, leading to experience-dependent recruitment of the motor cortex by behaviorally relevant sensory evidence.

### Recruitment of the motor cortex by sensory evidence prior to choice commitment

The localized nature of wide-field responses selective to pro-licking fast stimulus samples in the secondary and primary motor cortex is consistent with their recruitment by onset of the stimulus speed change when accounting for animals’ motor actions. These results contrast reports of widespread cortical activation during decision-making based on wide-field imaging (Allen et al., 2017; Pinto et al., 2019). Our findings are in agreement with systematic perturbations of activity across the cortex in a tactile discrimination task, which identified an area of the anterior motor cortex as uniquely required for preparation and execution licking responses (Guo et al., 2014). Furthermore, the rat homolog of the secondary motor cortex, which includes the frontal orienting field (FOF), has also been proposed as a key locus in the evolution of orienting decisions (Erlich et al., 2011). In a task in which rats base their choices by integrating auditory signals over hundreds of milliseconds (Brunton et al., 2013), the FOF represents evolving behavioral choices (Hanks et al., 2015) and is required for task performance (Erlich et al., 2015). Inactivation studies indicate that the MOs plays an important role in expression of behavioral choices in perceptual tasks (Guo et al., 2014; Allen et al., 2017; Zatka-Haas et al., 2018). Sustained modulation of MOs activity by sensory input in our task reveals that the MOs is engaged even in the absence of overt motor responses and thus has a role beyond motor execution. Wide-field imaging cannot discern whether these responses are carried by one homogeneous population or by different populations with different time courses of activity (Harvey et al., 2012; Scott et al., 2017). The GP classification model revealed that animals’ choices were influenced by the average stimulus speed on a timescale of ∼1 s, and the sustained recruitment of the secondary motor cortex by fluctuations in the stimulus could provide the neural substrate supporting this behavioral strategy. These observations suggest that this area contributes to perceptual choices independent of sensory modality or motor readout, perhaps by representing an emerging plan of action or the animal’s belief about the state of the stimulus.

### Temporal expectation modulates recruitment of the motor cortex by sensory evidence

The expectation of when the stimulus speed might change influenced the animals’ behavior in the task. Wide-field imaging permitted us to survey the effects of temporal expectation on sensory processing across the dorsal cortex at the population level. Areas MOs and MOp responded preferentially to pro-licking stimulus fluctuations during periods when stimulus speed change was likely. On the other hand, the initial wide-field responses in sensory areas were not modulated by expectation, contrary to earlier studies in rodents (Shuler and Bear, 2006; Jaramillo and Zador, 2011). This observation suggests that expectation does not systematically modulate their responses, although the bulk nature of wide-field imaging could have masked bidirectional modulation of individual neurons. Modulation of wide-field responses in the primary visual cortex did emerge later, several hundred milliseconds after stimulus presentation, perhaps as a consequence of top-down feedback. However, this modulation of visual cortical responses by expectation was in part explained by subtle differences in overt behavior (Figures S11E and S11F).

These results suggest that temporal expectation may influence the flow of sensory evidence from visual to motor areas. Although emergence of such selective transmission with learning has been implied previously (Makino et al., 2017), our results suggest that it can also be modulated dynamically and is not explained by differences in overt movements. A key question is how task-relevant inputs in the visual cortex are relayed to motor areas. Our experiments cannot disambiguate whether these signals are transmitted through direct corticocortical or indirect subcortical pathways that might be modified during learning. Projections from the sensory cortex to the basal ganglia have been implicated in acquisition and execution of perceptual tasks (Znamenskiy and Zador, 2013; Ruediger and Scanziani, 2020). A cortico-basal ganglion loop may play a similar role in our task by relaying task-relevant visual signals to motor areas in an expectation-dependent manner.

### Widespread movement-related modulation of the dorsal neocortex

We found that choice execution had a global influence on wide-field fluorescence. Even prior to lick detection, movements recorded by the body camera were accompanied by widespread recruitment of the dorsal cortex, masking neural signals underlying processing of the sensory stimulus. Such global influence of motor behavior on cortical activity is consistent with recent reports (Allen et al., 2017; Musall et al., 2019; Steinmetz et al., 2019; Stringer et al. 2019). However, the extent to which these global signals arise as the result of preparation or execution of movements or of sensory feedback associated with them remains unclear. If these signals indeed represent a form of efference copy and broadcast the selected motor action throughout the cortex, then they may serve as a substrate for reinforcement learning (Fee, 2014).

The ubiquity and magnitude of movement-related signals poses a major challenge for interpretation of neurophysiological signals when motor behavior is controlled or recorded inadequately. This challenge is illustrated by the version of the change detection task in which mice were free to run on the wheel during presentation of the baseline stimulus. The extended baseline period and stimulus design in our task provide a way to capture neural activity resulting from the interaction of sensory signals and animals’ expectation while controlling for global modulations associated with lick responses and other overt movements. The stark differences in the patterns of wide-field responses underlying processing of behaviorally relevant sensory signals and those arising during and prior to execution of behavioral choices highlight the importance of task designs that disambiguate these related and often concurrent processes.

By controlling for movement-related signals through task design and post hoc analyses, our results reveal how population activity across the mouse neocortex is modulated by the interaction of sensory evidence and prior knowledge and highlight the secondary motor cortex as a key region for further studies aimed at addressing neural mechanisms of distributed decision-making.

## STAR★Methods

### Key resources table

**Table:** 

REAGENT or RESOURCE	SOURCE	IDENTIFIER
**Experimental models: organisms/strains**		

Mouse: Camk2a-tTA	The Jackson Laboratory	JAX#007004
Mouse: tetO-GCaMP6s	The Jackson Laboratory	JAX#024742

**Software and algorithms**		

ScanImage, v5.7.0	Pologruto et al., 2003; Vidrio Technologies, LLC.	http://scanimage.vidriotechnologies.com/display/SIH/ScanImage+Home;jsessionid=7B68EEAE8A324DAC3FC67B48B636C75B
LabView 13, 17	National Instruments	https://www.ni.com/en-gb/shop/labview.html
MATLAB, 2017b, 2019b	Mathworks	https://www.mathworks.com/products/matlab.html
PsychToolbox-3	Kleiner et al., 2007	http://psychtoolbox.org/download
Suite2p	Pachitariu et al., 2016	https://github.com/MouseLand/suite2p
Baking Tray	https://github.com/SainsburyWellcomeCentre/BakingTray	https://doi.org/10.5281/zenodo.3631610
StitchIt	Han et al., 2018; https://github.com/SainsburyWellcomeCentre/StitchIt.	https://doi.org/10.5281/zenodo.3941901
Allen Brain Atlas API, CCF3, v3	Allen Institute for Brain Science	http://atlas.brain-map.org/
Code for optimization and analysis of Gaussian process models of mouse behavior	This paper	https://github.com/znamlab/rt_model_orsolic
Code for asymmetric Student-t model for neuropil correction	This paper	https://github.com/BaselLaserMouse/ast_model

**Deposited data**		

Behavioral datasets and trained models	This paper	https://doi.org/10.6084/m9.figshare.13606583.v1

### Resource availability

#### Lead contact

Further information and requests for resources and reagents should be directed to Thomas D. Mrsic-Flogel (t.mrsic-flogel@ucl.ac.uk).

#### Materials availability

This study did not generate new unique reagents.

#### Data and code availability

Behavioral data and code for optimization and analysis of the GP classification models of mouse behavior can be found at https://doi.org/10.6084/m9.figshare.13606583.v1 and https://github.com/znamlab/rt_model_orsolic. Due to the large size of the imaging dataset, the raw data have not been deposited in a public repository but will be made available upon request.

### Experimental model and subject details

All experiments were conducted in accordance with institutional animal welfare guidelines licensed by the Swiss cantonal veterinary office or the United Kingdom Home Office. To express calcium indicator in excitatory cells throughout the cortex, we crossed heterozygous Camk2a-tTA (JAX#007004) and homozygous tetO-GCaMP6s (JAX#024742) mice (Wekselblatt et al., 2016). We used 11 adult male mice (84-104 days old) for widefield imaging and 7 adult male mice (93-168 days old) for two-photon imaging.

### Method details

#### Animal housing and surgical procedures

Two weeks before the start of behavioral training, mice were switched to reversed light-cycle. Standard environment enrichment was provided in the form of a running wheel and cardboard tunnels or running wheel, clear tubes and wooded toys. After acclimatization, animals underwent surgery to prepare them for behavioral training and imaging.

Animals in the widefield cohort were anaesthetized with a mixture of fentanyl (0.05 mg per kg), midazolam (5.0 mg per kg), and medetomidine (0.5 mg per kg). Buprenorphine (0.1 mg per kg) and Enrofloxacin (5 mg per kg), were administered toward the end of the surgery, and during recovery. The skull was exposed and cleaned, and a metal head plate was secured to the skull around the edge of the occipital plate and the superior temporal line using dental cement (Super-Bond C&B, Sun Medical). The exposed imaging site was covered with transparent dental cement (Polymer L-Type Clear, Sun Medical) and a glass coverslip (150 um thickness), pre-cut using a diamond scribe to match the exposed surface of the skull (Silasi et al., 2016). A custom-made 3D printed light shield was then cemented to the preparation.

Animals in the two-photon cohort were anaesthetized using isoflurane anesthesia (1 - 4%). Dexamethasone (2 – 3 mg per kg) and carprofen (5 mg per kg) were administered prior to surgery. A head plate was implanted as described for widefield preparation, after which a craniotomy was performed and a round glass coverslip (3 mm) was implanted, centered ∼1.5-1.7 mm anterior and ∼0.5-0.7 mm lateral from Bregma.

#### Behavioral setup

Behavioral setups, similar to those previously described (Poort et al., 2015), were placed in sound isolated boxes. The mouse was head-fixed and placed on a polystyrene wheel (20 cm diameter, 12 cm width). Wheel movements were monitored using a rotary encoder (1000 pulses per revolution, Kübler) coupled to the wheel axle. Two 21.5” monitors were placed on each side of the animal (∼20 cm away from the animal, slightly angled and tilted toward animal’s body), covering approximately 100x70 degrees of visual space. Monitors were gamma-corrected with maximum luminance of ∼40 cd/m^2^ (Konica Minolta, LS-100 Luminance Meter). Custom written software in MATLAB controlled stimulation using PsychToolbox-3 (Kleiner et al., 2007). Soy milk rewards were delivered through the spout in front of the animal. Reward delivery was regulated via a solenoid pinch valve (NResearch). The spout was coupled to a piezo element whose output was used to measure the animal’s licking. Custom electronic hardware was used to amplify the piezo signals and control the valve. An air tube was placed ∼2 cm from the animal’s right cheek to deliver light air puffs (200 ms, 2 bar pressure, tip was cut open to 2 mm). The animal’s right eye was imaged with a CMOS camera (Imaging Source, 30 Hz) in order to track eye movements and pupil diameter. A second camera was placed in front of the animal capturing its body movements. To increase the throughput of behavioral training, animals were trained in parallel on 5 different setups (8 different setups for the two-photon cohort). Animals were assigned to the setups randomly from session to session. Behavioral data were acquired using custom-written code in LabView (National Instruments) and a PCI-6320 acquisition card (National Instruments).

#### Behavioral task

Each trial began with a gray isoluminant screen. After a randomized delay (minimum 3 s + sample from an exponential distribution with the mean 0.5 s) the baseline stimulus appeared (sinusoidal grating with the spatial frequency of 0.04 cycles per degree, square patch aperture equivalent to 3 grating periods, the direction of drift was randomized between upward or downward drift). The temporal frequency of the baseline stimulus increased after a randomized baseline period. Change times were sampled from an exponential distribution with a mean of 4 s truncated at 5 s and added to an offset of 3 s in early blocks and 10.5 s in late blocks. Initially in the widefield cohort, the offset for early probes (early changes that occur in late block) was 4 s (29/109 sessions), in rest of the sessions it matched the offset of the early block distribution. Removing trials outside of the 4-8 s overlap window during these sessions did not affect our conclusions in Figure 1J. On noisy trials, temporal frequency of the grating was drawn every 50 ms (3 monitor frames) from a lognormal distribution, such that log_2_-transformed TF had the mean of 0 and standard deviation of 0.25 octaves and the geometric mean TF on noisy trials was 1 Hz. In a subset of trials (30%) no noise was added and the baseline stimulus had a constant TF of 1 Hz. Mice were trained to report increases in mean temporal frequency by licking the spout to trigger reward delivery (drop of soy milk). If mice did not lick within 2.15 s from the change, the trial was a miss trial. If mice licked before the change happened, they received an air puff to the cheek. Responses in the first 150 ms (“refractory licks,” 58/19734 trials in widefield and 36/8901 trials in two-photon cohort) were not rewarded and were excluded from analysis of hit trials. In stationary mice, baseline stimulus was aborted if when movement exceeding 2.5 mm in a 50 ms window in either direction was detected.

#### Behavioral training

Before animals underwent training on the temporal frequency change detection task, several pre-training steps were taken in order to habituate the animal to the setup. One week after the surgery, mice were food-restricted and behavioral training started. Animals were handled for a minimum of 3 sessions, until mice were comfortable with the experimenter and were climbing on the experimenter’s hand while being given drops of soy milk. Animals were then introduced to short manual restraint periods in a soft cloth after which they were given soy milk rewards. Next, animals were head-fixed and placed on the running wheel of the behavioral training setup (10 – 20 min) with the monitors turned off and were trained to run on the wheel for reward. This step typically took 4 sessions. Two mice were not trained further than this step and were assigned to the naive cohort of the widefield experiments.

Next, to ensure that the animals understood the relationship between the stimulus presented on the monitor and reward availability, mice were pretrained on a simple task, where they had to lick in response to a change in the orientation of the grating. At this stage, translation of the grating was linked to the running speed of the mouse. As soon as mice started responding to the change in grating orientation, this step was complete. One mouse, which failed to learn to respond to orientation changes, was not trained further and was added to the naive cohort. Eight widefield mice proceeded training on the temporal frequency change detection task. Two of these mice were excluded from study due to lack of progress (too high abort rate due to early licks). It took the remaining six mice 14-21 sessions to acquire the task. Mice were initially allowed to run during the task. After observing strong modulation of cortical activity associated with running, mice were required to be stationary during the task.

Mice used for two-photon imaging were trained in the stationary version of the temporal frequency change detection task directly and acquired the task in 8-12 sessions (6 mice) and 34 sessions (1 mouse).

#### Widefield calcium imaging

Widefield calcium imaging was carried out using a custom-built tandem-lens epifluorescence macroscope using two photographic lenses (85mm f/1.8D objective, 50mm f/1.4D tube lens, Nikon) placed in face-to-face orientation (Ratzlaff and Grinvald, 1991). Excitation light from two LEDs: 470 nm (M470L3, Thorlabs, with excitation filter FF02-447/60-25, Semrock), and 405 nm (M405L3, Thorlabs, with excitation filter FF01-405/10-25, Semrock) was combined using a dichroic (FF458-Di02-25x36, Semrock) and delivered in Koehler configuration through a dichroic mirror (FF495-Di03, Semrock) placed in the infinity focused imaging path. Average power was ∼0.05 mW/mm^2^, similar to that in other studies (Wekselblatt et al., 2016). Images were acquired using an emission filter (525/50-25, Semrock) and an sCMOS camera (pco.edge 5.5, PCO) at 50 Hz in rolling shutter mode and binned on the fly 2x2 using manufacturer software. This lens combination resulted in a resolution of ∼20 μm per pixel. Excitation wavelengths were temporally interleaved by a microcontroller (Teensy 3.2) triggered by the camera rolling shutter exposure output. To avoid rolling shutter artifacts and crosstalk between 470 nm and 405 nm excitation frames, illumination was restricted to periods when all the lines being acquired corresponded to the same imaging frame (t_global_ in manufacturers’ documentation). A photodiode (PDA100A-EC, Thorlabs) recorded the onset of each visual stimulus frame to ensure precise alignment between visual stimulation and imaging data.

#### Two-photon calcium imaging

Two-photon calcium imaging was conducted using a custom-built resonant scanning two-photon microscope (INSS, UK) with a 16x water-immersion objective (NA 0.8, Nikon), at 930 nm excitation wavelength, ∼50 mW of power (Mai Tai, SpectraPhysics). GCaMP fluorescence was captured through a 520/40 emission filter (ET520/40 m, Chroma). Single imaging planes of 512 × 512 pixels, capturing a field of view of ∼440 × 440 μm, were acquired at ∼30 Hz using ScanImage v5.7.0 (Pologruto et al., 2003). To avoid cross-talk between imaging and visual stimulation, the monitor backlight was synchronized to the turnaround of the resonant mirror (Leinweber et al., 2014). A photodiode (PDA100A-EC, Thorlabs) recorded the onset of each visual stimulus frame to ensure precise alignment between visual stimulation and imaging data. At the end of the imaging session a Z stack was acquired capturing the surface of the brain and revealing the surface vasculature used for site localization. We imaged 7 mice, 34 sessions, at depths spanning 200 – 330 μm from the brain surface, at various locations over the secondary motor cortex.

#### Registration to Allen CCF reference atlas

At the end of the imaging experiments, mice from the widefield cohort were anaesthetized and five DiI (Invitrogen D3911) tracks were made across the imaging site by inserting a glass micropipette coated with DiI. Locations of the DiI tracks were recorded under the widefield macroscope to ensure that imaging frames could be successfully registered to *ex vivo* brain volumes. However, since we found that blood vessel patterns could be reliably reconstructed from *ex vivo* data, DiI tracks were not used for *ex vivo* / *in vivo* registration.

The mice were then anaesthetized with sodium pentobarbital and transcardially perfused with 4% paraformaldehyde. The brains were extracted, post-fixed overnight in 4% paraformaldehyde, and stored in 50 mM phosphate buffer. Brains were coronally sectioned (100 or 80 μm steps) and imaged at two optical planes per physical section resulting in voxel size of 1.32 × 1.32 × 50 μm or 2 × 2 x 40 μm using a custom serial two-photon tomography microscope. After illumination correction and image stitching, brain volumes were registered to the Common Coordinate Framework provided by the Allen Institute for Brain Science (CCF, v3 © 2015 Allen Institute for Brain Science, Allen Brain Atlas API, available from https://portal.brain-map.org/api/index.html) using Elastix (Klein et al., 2010) by applying rigid affine transformation followed by non-rigid deformation as previously described (Han et al., 2018).

To reconstruct the superficial blood vessel pattern from serial two-photon tomography volumes, we first identified the dorsal surface of the volume as the locations of the first voxel crossing a manually selected brightness threshold. We smoothed the location values with a median filter and used the fluorescence of the voxels near the surface to reveal blood vessels (Figure S4). Center locations of the two-photon imaging sites were manually determined based on the vasculature patterns of *in vivo* imaging sites and *ex vivo* stacks transformed to CCF atlas coordinates. Widefield imaging sites were first aligned to the reference imaging session for each mouse, and then aligned to the *ex vivo* stack transformed to CCF atlas coordinates by affine transformation based on manually selected control points.

### Quantification and statistical analysis

#### Behavioral data analysis

In the widefield cohort we included all the sessions after mice crossed the threshold of detecting more than 80% easiest changes in no-noise trials and interrupted less than 55% of no-noise trials due to early licking. Average detection rate across sessions for the easiest change was 96.9 ± 9.3% (mean ± sd), average early lick rate 19.4 ± 16.3% (mean ± sd). We excluded 6/115 sessions due to high abort rate due to movement. In the remaining sessions the average abort rate due to movement was 43.4 ± 17.8% (mean ± sd) where 52% of all movement induced aborts happened during the first 3.5 s of the stimulus. For two-photon imaging data analysis we included sessions where performance on two easiest changes was larger than 80% (19/34 sessions). Average detection rate across these sessions for the easiest change was 98% ± 0.2% (mean ± sd), average early lick rate 41.8% ± 21.7%, average movement abort rate 25.1% ± 20.7%.

When computing behavioral performance, all error bars are 95% confidence intervals, unless otherwise stated. For psychometric curves and hazard rates, confidence intervals were estimated using *binofit* in MATLAB, for chronometric curves they were calculated as the 0.025 and 0.975 quantiles of 2000 bootstrap samples with replacement.

To estimate hazard rates, the number of early licks and changes in one second bins was normalized by the total number of trials, excluding trials where early lick or change have already happened, or trial was aborted due to movement prior to the start of the bin.

To compute lick triggered averages, stimuli preceding early licks were averaged across animals, revealing mean stimulus information content prior to the lick. Confidence intervals were estimated by resampling early licks (2000 bootstrap samples with replacement).

#### GP classification model

A Gaussian process classification model (Rasmussen, 2006) was trained to predict the lick hazard rate in discrete time samples corresponding individual TF fluctuations (50 ms). The formulation and implementation of the model are described in detail below. In brief, the model received as its inputs the history of the visual stimulus over the past 50 samples (2.5 s) and time elapsed since the start of the trial. The stimulus history was filtered by multiplying the stimulus vector with a filter matrix, whose columns define the stimulus features that best predict mouse behavior (Vivarelli and Williams, 1999; Snelson and Ghahramani, 2006). The effective dimensionality of the filtered stimulus space was controlled by placing a hierarchical Gaussian prior on each column of the filter matrix, shrinking superfluous projections to 0 (Bishop, 1999; Beal, 2003). The time input was passed through a non-linear monotonic warping function parametrized as a sum of tanh functions (Snelson et al., 2004), to account for non-stationary nature of timing behavior (Gibbon, 1977) and excess early licks immediately following the onset of the baseline stimulus (Figure 1I). Filtered stimulus history and warped time served as inputs to the GP component of the model, whose output predicted the log-odds of licking. The covariance of the GP prior was defined as the sum of Matérn 5/2 kernels on filtered stimulus and warped time. To jointly fit behavior in different hazard rate blocks and across running and stationary versions of the task, the model was extended to include a hierarchy across experimental conditions (Hensman et al., 2013) by modifying the kernel to include population and hazard block-specific components. Computer code for model optimization and analysis of model fits can be found at https://github.com/znamlab/rt_model_orsolic.

#### Model structure

We aim to predict whether and when the mouse would lick in response to the visual stimulus on individual trials of the task. Since the trial is terminated once the mouse licks, we accomplish this by modeling the lick hazard rate – the probability of licking conditioned on the fact that the mouse has not licked up to that point during the trial. If we discretize time during each individual trial into “samples,” we will only have the opportunity to observe a lick in the ith sample of a trial, if there have been no licks in each of the i−1 preceding samples. Therefore, we will represent each trial outcome as the vector y – a series of zeros terminated by 1 or 0 depending on whether the mouse licked on the particular trial. The likelihood of observing a particular trial outcome y is the product of conditional likelihoods over the whole trial:

(1) $$\begin{array}{l} p\left(y|X)=p(y_{n}|y_{n-1}=0,x_{n}\right)\overset{n-1}{\underset{i=2}{\prod }}p\left(y_{i}=0|y_{i-1}=0,x_{i})\;p(y_{1}=0|x_{1}\right), \end{array}$$

given the design matrix $X=\left[x_{1},\ldots ,x_{n}\right]$ containing inputs over the course of the trial. Each input vector $x_{i}$ combines the stimulus history in the Q preceding samples and the time elapsed since the stimulus onset:

(2) $$\begin{array}{l} x_{i}={\left[s_{i},s_{i-1},\ldots ,s_{i-Q+1},t_{i}\right]}^{⊤}. \end{array}$$

For convenience, we sample the behavior every 50 ms – the duration of individual stimulus fluctuations in the task.

To capture the relationship between the inputs X and behavior observations y, we assume a latent function f representing the log-odds of licking. Therefore, the lick hazard rate at the ith moment in time is the logistic function of $\text{f}\left(x_{i}\right)$:

(3) $$\begin{array}{l} p\left(y_{i}=1|y_{i-1}=0,x_{i})=p(y_{i}=1|y_{i-1}=0,\text{f}\left(x_{i}\right)\right)=\frac{1}{1+\exp\left(-\text{f}\left(x_{i}\right)\right)}. \end{array}$$

In order to minimize assumptions on f, we do not impose a specific parametrization of f but introduce a prior distribution over functions, using a Gaussian process (GP) with a mean function m(x) and covariance function $\kappa \left(x,x^{\text{′}}\right)$:

(4) $$\begin{array}{l} \text{f}\left(x\right)∼GP\left(m\left(x\right),\kappa \left(x,x^{\text{′}}\right)\right). \end{array}$$

The GP prior distribution implies that the log-odds of licking at different moments in time during the session are jointly Gaussian, with the covariance defined as a function the stimulus immediately preceding each moment and time since trial onset:

(5) $$\begin{array}{l} p\left(\text{f}\left(x_{i}\right),\text{f}\left(x_{j}\right)\right)=N\left(\left[\begin{array}{l} m\left(x_{i}\right)\\ m\left(x_{j}\right) \end{array}\right],\left[\begin{array}{ll} \kappa \left(x_{i},x_{i}\right) & \kappa \left(x_{i},x_{j}\right)\\ \kappa \left(x_{j},x_{i}\right) & \kappa \left(x_{j},x_{j}\right) \end{array}\right]\right)\;\forall i,j\in \left[1;n\right]. \end{array}$$

In this framework, given a training dataset $D={\left\{\left(x_{i},y_{i}\right)\right\}}_{i=1}^{N}$ aggregating all samples for the training trials, we can predict the mouse behavior at any sampled time point n of a test trial using the predictive distribution:

(6) $$\begin{array}{l} p\left(y_{\text{∗}}|X_{\text{∗}},D\right)=\int p\left({y_{\text{∗}}}_{n}|{y_{\text{∗}}}_{n-1}=0,{f_{\text{∗}}}_{n}\right)\overset{n-1}{\underset{i=2}{\prod }}p\left({y_{\text{∗}}}_{i}=0|{y_{\text{∗}}}_{i-1}=0,{{\text{f}}_{\text{∗}}}_{i}\right)\;p\left({y_{\text{∗}}}_{1}=0|{{\text{f}}_{\text{∗}}}_{1}\right)\;p\left(f_{\text{∗}}|D\right)\;df_{\text{∗}} \end{array}$$

where $X_{\text{∗}}=\left[{x_{\text{∗}}}_{1},\ldots ,{x_{\text{∗}}}_{n}\right]$ is the test input data, $f_{\text{∗}}={\left[{{\text{f}}_{\text{∗}}}_{1},\ldots ,{{\text{f}}_{\text{∗}}}_{n}\right]}^{⊤}={\left[\text{f}\left({x_{\text{∗}}}_{1}\right),\ldots ,\text{f}\left({x_{\text{∗}}}_{n}\right)\right]}^{⊤}$ the vector of corresponding latent function values and $y_{\text{∗}}={\left[{y_{\text{∗}}}_{1},\ldots ,{y_{\text{∗}}}_{n}\right]}^{⊤}$ the behavior whose probability is evaluated.

The next section will describe how to evaluate the posterior distribution of f and what it implies in terms of training. The approach is based on the work of Hensman, et al. (2015) and is summarized here for the sake of completeness and reproducibility. The subsequent sections will describe the kernel and mean functions and summarize specifics of the implementation.

#### GP posterior estimation

The posterior distribution can be rewritten as an integral of two terms:

(7) $$\begin{array}{l} p\left(f_{\text{∗}}|D\right)=\int p\left(f_{\text{∗}}|f\right)\;p\left(f|D\right)\;d\text{f}, \end{array}$$

where $f=\left[{\text{f}}_{1},\ldots ,{\text{f}}_{N}\right]$ is the vector of latent function values on the training data. The first term is the conditional distribution of the latent function values for the test data given the latent function values of the training data. As a property of the GP prior, we can analytically derive its form as the density of a multivariate normal distribution, that we note $p\left(f_{\text{∗}}|f\right)=N\left(\mu_{\text{∗}},\Sigma_{\text{∗}}\right)$. The mean and covariance parameters $\left(\mu_{\text{∗}},\Sigma_{\text{∗}}\right)$ depend on the covariance of the training data ${\left[K_{ff}\right]}_{i,j}=\kappa \left(x_{i},x_{j}\right)$, and the covariance of the test data with the training data ${\left[K_{\text{∗}f}\right]}_{i,j}=\kappa \left({x_{\text{∗}}}_{i},x_{j}\right)$:

(8) $$\begin{array}{l} \mu_{\text{∗}}=m\left(X_{\text{∗}}\right)+K_{\text{∗}f}K_{ff}^{-1}\left(f-m\left(X\right)\right)\;\mathrm{and}\;\Sigma_{\text{∗}}=K_{ff}-K_{\text{∗}f}K_{ff}^{-1}K_{\text{∗}f}^{⊤}, \end{array}$$

where $m\left(X_{\text{∗}}\right)={\left[m\left({x_{\text{∗}}}_{1}\right),\ldots ,m\left({x_{\text{∗}}}_{n}\right)\right]}^{⊤}$ and $m\left(X\right)={\left[m\left(x_{1}\right),\ldots ,m\left(x_{N}\right)\right]}^{⊤}$.

The second term p(f|D), the posterior of the latent function values on the training data, is more problematic. As our likelihood is not normally distributed (Equation 3, it cannot be expressed in closed-form; Rasmussen, 2006). We replace it with a normal variational approximating distribution $q\left(f\right)=N\left(\mu_{f},\Sigma_{f}\right)$. The mean and covariance parameters $\left(\mu_{f},\Sigma_{f}\right)$ are optimized to minize the Kullback-Leibler (KL) divergence between p(f|D) and q(f), a measure of discrepancy between the two distributions.

With this variational approximation, we can replace Equation 7 with an approximate posterior distribution:

(9) $$\begin{array}{l} p\left(f_{\text{∗}}|D\right)\approx q\left(f_{\text{∗}}|D\right)=\int p\left(f_{\text{∗}}|f\right)\;q\left(f\right)\;d\text{f}, \end{array}$$

which possesses a closed-form expression. Both terms in the integral being normal distribution density functions, the result is also a normal distribution density $q\left(f_{\text{∗}}|D\right)=N\left({\tilde{\mu }}_{\text{∗}},{\tilde{\Sigma }}_{\text{∗}}\right)$ with parameters defined as follows:

(10) $$\begin{array}{l} {\tilde{\mu }}_{\text{∗}}=m\left(X_{\text{∗}}\right)+K_{\text{∗}f}K_{ff}^{-1}\left(\mu_{f}-m\left(X\right)\right)\;\mathrm{and}\;{\tilde{\Sigma }}_{\text{∗}}=K_{ff}-K_{\text{∗}f}{\left(K_{ff}-\Sigma_{f}\right)}^{-1}K_{\text{∗}f}^{⊤}. \end{array}$$

For a given test input ${x_{\text{∗}}}_{i}$, the posterior mean can be rewritten as:

(11) $$\begin{array}{l} {{\tilde{\mu }}_{\text{∗}}}_{i}=m\left({x_{\text{∗}}}_{i}\right)+\sum_{j=1}^{N}\alpha_{i}\kappa \left(x_{\text{∗}},x_{j}\right)\;\mathrm{where}\;\alpha =K_{ff}^{-1}\left(\mu_{f}-m\left(X\right)\right). \end{array}$$

Making inferences using Equation 10 requires the entire training dataset D and becomes computationally intractable for large N. To reduce computational complexity, we replace the training data with a set of M (such that M≪N) pseudo input points, also called inducing points, $Z=\left[z_{1},\ldots ,z_{M}\right]$ and latent function values $u=\left[\text{f}\left(z_{1}\right),\ldots ,\text{f}\left(z_{M}\right)\right]$. In this scenario, the variational approximating distribution becomes q(f)=∫p(f|u)q(u)du where $q\left(u\right)=N\left(\mu_{u},\Sigma_{u}\right)$. With this new variational approximation, the parameters of the approximate posterior distribution in Equation 10 become:

(12) $$\begin{array}{l} \tilde{\mu }=m\left(X_{\text{∗}}\right)+K_{\text{∗}u}K_{uu}^{-1}\left(\mu_{u}-m\left(Z\right)\right)\;\mathrm{and}\;\tilde{\Sigma }=K_{uu}-K_{\text{∗}u}{\left(K_{uu}-\Sigma_{u}\right)}^{-1}K_{\text{∗}f}^{⊤}, \end{array}$$

where ${\left[K_{uu}\right]}_{i,j}=\kappa \left(z_{i},z_{j}\right)$ and ${\left[K_{\text{∗}u}\right]}_{i,j}=\kappa \left({x_{\text{∗}}}_{i},z_{j}\right)$. As a consequence, any computation involving these quantities only requires inverting a M×M matrix, rather than a N×N matrix.

In this final formulation, training a model consists in optimizing the values of the inducing points and values (Z,u) as well as the variational distribution parameters $\left(\mu_{u},\Sigma_{u}\right)$, in order to minimize the KL-divergence between p(f|D) and q(f). Minimizing the KL-divergence objective is equivalent to maximizing the model evidence lower bound (ELBO) defined as:

(13) $$\begin{array}{c} L=E_{q\left(f\right)}\left[log\;p\left(y|f\right)\right]-\text{KL}\left(\text{q}\left(u\right)\|\text{p}\left(u\right)\right)\\ =\sum_{i=1}^{N}E_{q\left(f\right)}\left[log\;p\left(y_{i}|y_{i-1},{\text{f}}_{i}\right)\right]-\text{KL}\left(\text{q}\left(u\right)\|\text{p}\left(u\right)\right). \end{array}$$

As the ELBO approximates the model marginal log-likelihood, it is also used to learn the model hyperparameters, i.e., the kernel function and mean function parameters. All parameters are optimized using a gradient descent technique. The definition of the ELBO involving a simple sum over the training data, we employ a stochastic version the gradient descent using only a random subset of the training data at each iteration.

#### Kernel and mean functions

In general the mean function m(x) can be set to 0 with no reduction in model performance, as the posterior distribution can capture the mean. However, as the mean log-odds of licking are far below 0 (mice do not lick for the vast majority model samples), we include a constant mean function to ensure that the model makes sensible predictions outside of the range of the training data.

We define the kernel function as the sum of stimulus and time dependent components:

(14) $$\begin{array}{l} \kappa \left(x,x^{\text{′}}\right)=\kappa_{s}\left(x,x^{\text{′}}\right)+\kappa_{t}\left(x,x^{\text{′}}\right), \end{array}$$

where $\kappa_{s}\left(x,x^{\text{′}}\right)$ and $\kappa_{t}\left(x,x^{\text{′}}\right)$ depend on stimulus history s or the time elapsed since the stimulus onset t, respectively. An advantage of the additive form of the kernel is that stimulus- and time-dependent components of the log-odds can be readily separated. The posterior predictive mean from Equation 11 can be decomposed into:

(15) $$\begin{array}{c} {\tilde{\mu }}_{*i}=m\left(x_{*i}\right)+\sum_{j=1}^{N}\alpha_{i}\left(\kappa_{s}\left(x_{*i},x_{j}\right)+\kappa_{t}\left(x_{*i},x_{j}\right)\right)\\ =m\left(x_{*i}\right)+\sum_{j=1}^{N}\alpha_{i}\kappa_{s}\left(x_{*i},x_{j}\right)+\sum_{j=1}^{N}\alpha_{i}\kappa_{t}\left(x_{*i},x_{j}\right)\\ =m\left(x_{*i}\right)+{\tilde{\mu }}_{is}+{\tilde{\mu }}_{it}. \end{array}$$

To identify stimulus features that best explain observed behavior, we first multiply the stimulus history by a Q×D matrix of filters W (Vivarelli and Williams, 1999; Snelson and Ghahramani, 2006), where Q is the number of stimulus history samples included in the model and D is the number of stimulus filters:

(16) $$\begin{array}{l} \varphi =s^{⊤}W. \end{array}$$

Our approach is to select an arbitrarily large D and control the effective dimensionality of the filtered stimulus space by placing an automatic relevance determination (ARD) prior on W (Bishop, 1999; Beal, 2003). The ARD prior assumes a zero-mean Gaussian prior on each column $w_{d}$ of the matrix W:

(17) $$\begin{array}{l} p\left(W|\nu \right)=\prod_{d=1}^{D}{\left(\frac{\nu_{d}}{2\pi }\right)}^{D/2}\exp\left(-\frac{\nu_{d}{w_{d}}^{2}}{2}\right), \end{array}$$

and a gamma distributed prior on the precision vector $\nu =\left[\nu_{1},\ldots ,\nu_{D}\right]$. This prior over ν favors high precisions, consequently shrinking the columns of W that do not contribute to the prediction performance of the model. To estimate the loadings of W, we infer an approximate posterior distribution over (W,ν) using the automatic differentiation variational inference (ADVI) framework (Kucukelbir et al., 2017), which extends the ELBO definition of the model (Equation 13) with additional terms.

We then use a Matérn 5/2 kernel over filter outputs φ such that $x={\left[\varphi_{1},\ldots ,\varphi_{D},t\right]}^{⊤}$. Since their magnitude can be adjusted by scaling the columns of W, the length scale of the stimulus kernel is fixed to 1 to avoid over-parametrizing the model. Therefore, $\kappa_{s}$ depends only on the euclidean distance L between φ and $\varphi^{'}$:

(18) $$\begin{array}{l} \kappa_{s}\left(x,x^{\text{′}}\right)=\sigma_{s}^{2}\left(1+\sqrt{5}L+5L^{2}/3\right)\exp\left(-\sqrt{5}L\right),\;\mathrm{where}\;L={\left|\left|\varphi -\varphi^{'}\right|\right|}_{2}. \end{array}$$

It is well established that precision of timing behavior is not constant but varies with the duration of time intervals (Gibbon, 1977). To account for this non-stationarity, we passed the input to the time component of the kernel through a non-linear monotonic warping function, parametrized as a sum of tanh functions (Snelson et al., 2004):

(19) $$\begin{array}{l} t_{w}=t+\sum_{k=1}^{J}a_{k}\tanh\left(b_{k}\left(t+c_{k}\right)\right). \end{array}$$

We optimize the parameters (a,b,c) during model training. The time kernel $\kappa_{t}$ is then computed as a Matérn 5/2 kernel over warped time with its own length scale and variance parameters:

(20) $$\begin{array}{l} \kappa_{t}\left(x,x^{\text{′}}\right)=\sigma_{t}^{2}\left(1+\frac{\sqrt{5}\left(t_{w}-t_{w}^{\text{′}}\right)}{\ell_{t}}+\frac{5{\left(t_{w}-t_{w}^{\text{′}}\right)}^{2}}{3\ell_{t}^{2}}\right)\exp\left(-\frac{\sqrt{5}\left(t_{w}-t_{w}^{\text{′}}\right)}{\ell_{t}}\right). \end{array}$$

#### Hierarchical structure

Our dataset contains trials recorded under different experimental conditions, such as blocks of trials with different distributions of stimulus change times, as well as running and stationary conditions. We aimed to extend the model to capture the differences in behavior between these experimental blocks, while also learning their shared features. The GP framework offers a simple and rigorous approach for dealing with such structured data (Hensman et al., 2013).

We introduce an indicator variable b, which denotes the experimental block for each sample, and split each part of the covariance $\kappa \left(x,x^{\text{′}}\right)$ into population and block-specific components:

(21) $$\begin{array}{l} \kappa \left(x,x^{\text{′}}\right)=\left\{ \begin{array}{cc} \kappa_{s_{p}}+\kappa_{s_{b}}+\kappa_{t_{p}}+\kappa_{t_{b}} & \mathrm{when}\;b=b^{\text{′}}\\ \kappa_{s_{p}}+\kappa_{t_{p}} & \mathrm{otherwise}. \end{array}\right. \end{array}$$

Population and block-specific covariance functions share the same forms, described by (18), (19), (20), and stimulus features defined by W but have their own hyperparameters, variances $\sigma_{s}^{2}$ and $\sigma_{t}^{2}$, and length scale $\ell_{t}$.

#### Model implementation and training

The model is implemented on the basis of the Stochastic Variational GP class of the *GPflow* Python package (de G. Matthews et al., 2017), which relies on *Tensorflow* (Abadi et al., 2015) for automatic differentiation and GPU-based computations.

Model training was carried out independently for each mouse, using behavioral data from both stationary and trained versions of the task. The block variable b (Equation 21) indicated whether a trial was acquired in the running task, or in early or late hazard rate blocks in the stationary task. The dataset was split into training (60%), validation (20%), and test (20%) sets, stratified according to change strength and experimental block.

We used 450 inducing points and assigned 150 to each experimental block. Inducing points were initialized using a mini-batch variant of the K-means clustering algorithm, provided by the *scikit-learn* Python package (Pedregosa et al., 2011). We set the number of stimulus history samples Q to 50, the number of filters D to 15, and the number of tanh functions J (Equation 19) to 5. Filter coefficients W and the parameters (a,b,c) were randomly initialized from a standard normal distribution.

We performed the optimization of all parameters using the Adam algorithm (Kingma and Ba, 2014) with the default settings and a learning rate of 0.001 employing 12000 samples per mini-batch. Computations took place on Nvidia GeForce 2070 RTX or 2080 RTX graphics cards, and were terminated after a maximum time of 10 hours or when convergence was reached as assessed by predictive performance on the validation set.

Once a model was fitted, we estimated the predictive distribution for each time point of each trial with Equation 6, replacing the integral with a Monte Carlo integral using 500 samples from the approximate posterior distribution. We did not use the full posterior distribution of W but replaced it with the posterior mean. To assess the performance of the model and capture uncertainty of its predictions, we generated behavior replicates by drawing licks from the predictive distribution for each trial in the test set. If a sampled lick occurred after the baseline period, the corresponding replicate trial was labeled as a hit and a reaction time was calculated as the time from change to the lick. We repeated this sampling procedure to obtain 500 replicates of the test dataset and generated psychometric and chronometric curves and the distribution of lick times for each replicate. Figure 2 and Figures S2 and S3 show the median, 2.5%, and 97.5% quantiles of the sampled curves. We also performed model comparisons based on their predictive performance, using the averaged predictive log-likelihood of trials in the dataset (Figure S3H).

To examine the effect of removing one of top two the stimulus filters in Figure S3, the corresponding column of the filter matrix W was set to 0 before evaluating model predictions. Performance of the full and ablated models was then compared by evaluating them 200 times over the entire dataset, including training, validation and test folds.

#### Neural data analysis

##### Pre-processing of widefield imaging data

Saved frames were checked for dropped frames and XY motion artifacts, binned 4x4 using a box kernel, and separated to 405 nm and 470 nm channels. The camera offset (average of 10000 dark frames) was removed from each frame. Each pixel in each session was low-cut filtered (cut-off at 0.00333 Hz), preserving the DC offset. For hemodynamic signal correction, 405 nm channel was linearly interpolated to time points of 470 nm channel frames by taking the average of 405 nm frames immediately before and after each 470 nm frame. The ratio of 470 and 405 channels was then normalized by the mean of the ratio:

$$f=\;\frac{f_{470}/f_{405}}{\frac{1}{N}\sum_{k}f_{470_{k}}/f_{405_{k}}}.$$

Imaging was conducted at 50 Hz, resulting in a hemodynamics-corrected frame rate of 25 Hz. Frames were further downsampled 2x resulting in 170 μm per pixel resolution. To correct for differences in illumination and prep quality across the imaging site within individual sessions, and across sessions and animals, fluorescence traces for each pixel were normalized by their standard deviation within each imaging session. Finally, normalized imaging frames were aligned to the Common Coordinate Framework provided by the Allen Institute for Brain Science (Figure S4).

##### Responses to task events

To compute fluorescence responses associated with the baseline stimulus onset, stimulus change, correct, and early licks, we first identified the imaging frame, which was being exposed when a given event occurred. This frame corresponds to time 0 in fluorescence traces in Figures 3 and 4. We then extracted fluorescence traces around each event. Aligned fluorescence and movement traces were then baseline corrected by subtracting the mean in 480 ms (for stimulus and change onset) or 2000 ms (for licks) prior to event onset. For ROI-based analyses, we first computed the mean fluorescence within a given ROI and then processed the ROI traces as described above.

To minimize the impact of behavioral responses, for stimulus onset traces in Figure 3, we only included frames which occurred during the baseline stimulus presentation, excluding frames acquired after change onset or occurring less 1 s prior to early licks or wheel movement in trained animals. If the resulting fluorescence trace was shorter than 0.5 s, the entire trial was excluded.

##### Responses to baseline TF fluctuations

We first resampled the TF of the baseline stimulus at the sampling rate of imaging acquisition. To do this, we computed the mean log-TF presented during each imaging frame acquired during the baseline stimulus, weighted by their presentation duration:

$$s=\frac{\sum_{i}p_{i}log_{2}v_{i}}{\sum_{i}p_{i}}.$$

$\nu_{i}$ is the TF of the i-th monitor frame presented during a given imaging frame, $\;p_{i}$ is its duration (16.7 ms or less, for monitor frames, which spanned two imaging frames).

For the regression analysis in Figures 5A, 5B, and 6D we evaluated the impact of stimulus samples s on future fluorescence responses $f_{t}$ by shifting the responses by time t (step = 50 ms, maximum shift = 2 s). To measure the change in fluorescence evoked by the stimulus sample, each shifted fluorescence vector $f_{t}$ was then corrected by subtracting the fluorescence at the time of the stimulus presentation f (0 ms lag). We then fit the following regression model relating baseline corrected fluorescence to log_2_-transformed TF for each time lag independently:

$$f_{t}-f\;=\;a_{t}+sb_{t}+\varepsilon_{t}.$$

$b_{t}$ quantifies the modulation of fluorescence responses by the stimulus, while the intercept term $a_{t}$ captures unrelated changes in the time course of fluorescence responses. We only included fluorescence frames acquired during the baseline stimulus and at least 1 s prior to early licks or wheel movements. Model coefficients and their confidence intervals were estimated using the function *regress* in MATLAB.

In expectation manipulation analysis in Figure 7A, the above model was fit for each block independently. To quantify the effects of temporal expectation manipulations, we added a predictor corresponding to the expectation block and an interaction term capturing the effect of the expectation block on the slope of the fluorescence/stimulus relationship to the regression model above:

$$f_{t}-f=a_{t}+sb_{t}+ec_{t}+e\circ sd_{t}+\varepsilon_{t}\;,$$

where the indicator variable e is 1 for samples from the early block and 0 for samples from the late block, and e∘s is an elementwise product between e and s. Regression coefficients $d_{t}$ corresponding to this interaction term estimate how expectation modulates fluorescence responses to fluctuations in the visual stimulus (Figure S11A), and determine periods in Figure 7A when this interaction term is significantly different from 0 (p < 0.05).

We then quantified the time course of regression coefficients in different cortical areas by fitting a multiexponential model:

bt=bmax(1−e−tτr)ze−tτd

Peak response $b_{max}$, power coefficient z, and rise and decay time constants $\tau_{r}$ and $\tau_{d}$ were optimized using *lsqnonlin* in MATLAB. The peak response in Figure 5C was directly given by the corresponding fit parameter. Response latency (Figure 5D) was estimated as the time lag, at which the multiexponential fit exceeded 50% of its maximum absolute value. The half decay time (Figure 5E) was estimated the as time following the response maximum, at which the fit, extrapolated if necessary, fell below 50% of its maximum value. If this did not occur within a 4 s window from the stimulus sample, half decay time is reported as not determined (N.D.).

For ridge regression in Figure S9, we included stimulus and widefield data from the same period as for the model in Figure 5, but now with additional continuous predictors of face and body movement videography, and running wheel movement. We then constructed a design matrix X,

$$X=\;\left[\;1\;S\;M_{\text{body}}\;M_{\text{face}}\;M_{\text{wheel}}\;\right],$$

where 1 is a column of ones, S is the history of the resampled sensory stimulus over the past 2 s, and $M_{\text{body}}$, $M_{\text{face}}$, and $M_{\text{wheel}}$ are movement predictors (overall body camera movement, face region, and wheel movement, respectively) over the past 2 s and 0.52 s into the future. Each predictor except for the constant column was rescaled by its standard deviation.

We used 5-fold cross-validation to estimate the optimal ridge penalty. To this end, the design matrix X and fluorescence vector f were divided into 5 equal contiguous blocks. For each fold, 4 of the blocks were assigned to the training set, while the remaining block was assigned to the test set. The block approach was used to avoid overfitting on timeseries data. Since the training and test blocks primarily contained data from different mice, it also encouraged selection of coefficients that generalized well across animals.

For each fold, we then estimated the regression coefficients $b_{\text{train}}$

$$b_{\text{train}}=\;{\left(X_{\text{train}}^{T}X_{\text{train}}+\lambda I\right)}^{-1}X_{\text{train}}^{T}f_{\text{train}},$$

where I is the identity matrix with zero replacing its first element to avoid regularizing the intercept of the model, and λ is the ridge penalty. We then evaluated the mean squared error for each fold, and selected the λ that minimized the average mean squared error across folds:

$$\frac{1}{n}{\left\|X_{\text{test}}b_{\text{train}}\;-\;f_{\text{test}}\right\|}_{2}^{2}.$$

This procedure was repeated selecting the optimal λ for every pixel by searching over 36 values logarithmically spaced between 10^−2^ and 10^5^. The optimal penalty was then used to estimate the ridge regression coefficients using the entire dataset:

$$b_{\text{ridge}}={\left(X^{T}X+\lambda I\right)}^{-1}X^{T}f.$$

Some pixels on the edges of the imaging prep were only imaged in a subset of mice. For the three most common combinations, we refit the model using the design matrix X and fluorescence vector f only including sessions acquired in the mice imaged for those pixels. This left 116/2211 pixels on the extreme edges of the prep which were not used for the ridge regression analysis. Ridge regression coefficients in the Figures were divided by the standard deviation of each predictor column to correct for rescaling of predictors prior to regression. Note that the coefficients of this regression model corresponding to baseline TF fluctuations are not directly comparable to those in Figure 5B, as the latter considered one time lag at a time and did not use the ridge penalty.

As a more stringent test of whether movements could explain widefield responses to TF fluctuations, we first used ridge regression to estimate the impact of movements alone and then analyzed the residuals of this model. Using the design matrix

$$X_{M}=\;\left[1\;M_{\text{body}}\;M_{\text{face}}\;M_{\text{wheel}}\right]$$

we estimated regression coefficients $b_{M}$following the procedure above. We then applied the same analysis as in Figure 5B to characterize the effect of temporal frequency fluctuations on widefield fluorescence, using the residuals of this regression model $f-\;X_{M}b_{M}$ in place of raw fluorescence values. We used the same approach to correct for movement in the analysis of interactions between TF fluctuations and expectation in Figure S11.

For the analyses of responses to binned TF fluctuations in Figures 5F, 5G, 7B and 7C, we computed mean fluorescence traces aligned to resampled TF fluctuations within each TF bin, again only including fluorescence frames acquired during the baseline stimulus and at least 1 s prior to early licks or wheel movements. To account for the overall time course of the baseline stimulus response (Figure 3), we then subtracted the mean response to the middle bin from responses to extreme bins. Due to the large sample size (tens to hundreds of thousands of imaging frames), confidence intervals were computed using the Normal approximation from the standard errors of mean fluorescence responses in each bin.

As for analyses of responses to task events, to quantify ROI responses to baseline TF fluctuations, we first computed the mean fluorescence within a given ROI and then analyzed the ROI traces as described above.

##### Responses to stimulus changes

For the ridge regression model of responses during the change period (Figures 4H–4J) we constructed a design matrix X,

$$X=\;\left[1\;C_{1}\;C_{1.25}\;C_{1.35}\;C_{1.5}\;C_{2}\;C_{4}\;M_{\text{body}}\;M_{\text{face}}\;M_{\text{wheel}}\;L\right],$$

where 1 is a column of ones, $\;C_{s}$ are matrices of categorical predictors corresponding to onsets of changes of s Hz, time lagged over 2 s. $M_{\text{body}}$, $M_{\text{face}}$, and $M_{\text{wheel}}$ are matrices of continuous movement predictors (overall body camera movement, face region, and wheel movement) over the past 2 s and 0.52 s into the future, while L is a categorical predictor matrix corresponding to times of licks, time lagged over 0.68 s past and up to 0.52 s into the future.

To account for fluctuations in fluorescence preceding change onset, fluorescence responses on individual trials were corrected by the fluorescence value at the time of change onset before being assembled into the fluorescence vector f. The model was then fit using ridge regression with 5-fold cross validation as described above for the analysis of baseline responses.

The vector of regression coefficients was then subdivided into components corresponding to different predictors:

$$b_{\mathrm{ridge}}=\;\left[b_{1}\;b_{C_{1}}^{T}\;b_{C_{1.25}}^{T}\;b_{C_{1.35}\;}^{T}\;b_{C_{1.5}}^{T}\;b_{C_{2}}^{T}\;b_{C_{4}}^{T}\;b_{M_{\text{body}}}^{T}\;b_{M_{\text{face}}}^{T}\;b_{M_{\text{wheel}}}^{T}\;b_{L}^{T}\right]$$

To highlight components of the response evoked by the change in the sensory stimulus and not other time-dependent fluctuations in fluorescence, Figure 4H depicts coefficients corresponding to different change strengths corrected by coefficients corresponding to no-change trials, i.e., $b_{C_{s}}-b_{C_{1}}$.

##### Analysis of two-photon imaging data

Two-photon imaging frames were motion corrected and segmented using suite2p software https://github.com/MouseLand/suite2p; Pachitariu et al. 2016). Cell and non-cell ROIs were manually curated for each session, and non-cells were further filtered based on their size (non-cell ROIs larger than 1/3 of the largest cell ROI were excluded), aiming to include mostly dendrites in this category. However, since we did not confirm each ROI as dendrite, we refer to this category as neurites.

ROI and surrounding neuropil traces were detrended by subtracting the rolling 10^th^ percentile in a 4000 frame (∼2 minute) window, and somatic and neurite traces were corrected for neuropil contamination using the ASt algorithm (https://github.com/BaselLaserMouse/ast_model). The ASt algorithm fits both ROI and surround fluorescence to asymmetric Student-t (ASt) distributions, whose mean was determined by a common neuropil signal contributing to both ROI and surrounding fluorescence. The ASt distribution has different degrees of freedom $v_{1}$ and $v_{2}$ for its left and right tails. We set $v_{1}=30$ and $v_{2}=1$, such that the left tail was approximately Gaussian, while the right tail resembled the Cauchy distribution. Thus the model allows for large positive but not negative deviations, consistent with the nature of calcium fluorescence signals. The advantage of this approach over widely used methods, which involve directly subtracting the scaled surrounding fluorescence signal from the ROI fluorescence trace, lies in the use of the ASt distribution to model deviations in both ROI and surround signals. The long right tail of the ASt distribution helps prevent over-estimating the neuropil component for densely active cells. At the same time, the use of the ASt distribution for the surround signal helps account for transient increases in fluorescence arising from unannotated neurites or cell bodies, which could otherwise result in false negative transients in the corrected trace.

For comparison of two-photon and widefield calcium signals in Figures 6D and 6E, we calculated the mean fluorescence within each two-photon ROI category (cells, neurites, neuropil) by averaging all z-scored detrended traces within each category. We then repeated the same regression analysis as in Figure 5A. For the analysis in Figure 6E, the resulting two-photon traces (regression coefficients across different time lags) were resampled to the widefield framerate using linear interpolation. We then computed the Pearson correlation between the time courses of responses (0-1.48 s) of different two-photon ROIs and the widefield signal. We also calculated the correlation of individual mice from the widefield cohort to the average widefield response of the remaining mice. For the analyses of responses to binned TF fluctuations in Figure 6F, same analysis was repeated as for Figures 5F-G.

#### Videography data extraction

The right eye was illuminated with a custom-made IR-light source and imaged using a CMOS camera (DMK22BUC03, Imaging Source, ∼30 Hz). Frames were filtered using a 2D Gaussian filter (σ = 2) and thresholded to identify low IR light reflectance areas (< 7.5% image max intensity). Regions were filtered based on circularity (perimeter squared to area ratio < 1.6 × 4π) and size (> 100 pixels). Edges of each region were detected using canny method and filtered using a Gaussian filter (σ = 1). An ellipse was fitted iteratively to the region matching the criteria by minimizing the geometric distance between the area outline and the ellipse using nonlinear least-squares (Brown 2018). Pupil diameter was estimated as the major axis of the ellipse after z-scoring within each session to correct for differences in illumination or camera position.

A second CMOS camera was placed in front of the animal capturing animal’s face and body. Body motion was expressed as the mean squared difference between the two consecutive frames z-scored within each session. Video frames were cropped such that the implant was not included, to avoid artifacts related to interleaved wavelength excitation. To measure fine facial movements, we have created a separate ROI (100 pixels square), centered on the mouse’s nose.

## Supplemental information

Document S1. Figures S1–S11

Document S2. Article plus supplemental information

## References

[bib1] Abadi M., Agarwal A., Barham P., Brevdo E., Chen Z., Citro C., Corrado G.S., Davis A., Dean J., Devin M. (2015). TensorFlow: Large-scale machine learning on heterogeneous systems. arXiv.

[bib2] Allen W.E., Kauvar I.V., Chen M.Z., Richman E.B., Yang S.J., Chan K., Gradinaru V., Deverman B.E., Luo L., Deisseroth K. (2017). Global Representations of Goal-Directed Behavior in Distinct Cell Types of Mouse Neocortex. Neuron.

[bib3] Andermann M.L., Kerlin A.M., Roumis D.K., Glickfeld L.L., Reid R.C. (2011). Functional specialization of mouse higher visual cortical areas. Neuron.

[bib4] Beal M.J. (2003). Variational algorithm for approximate Bayesian inference.

[bib5] Bishop C.M., Kearns M., Solla S., Cohn D. (1999). Bayesian PCA. Advances in Neural Information Processing Systems.

[bib6] Brown R. (2018). fitellipse.m. https://www.mathworks.com/matlabcentral/fileexchange/15125-fitellipse-m.

[bib7] Brunton B.W., Botvinick M.M., Brody C.D. (2013). Rats and humans can optimally accumulate evidence for decision-making. Science.

[bib8] Burgess C.P., Lak A., Steinmetz N.A., Zatka-Haas P., Bai Reddy C., Jacobs E.A.K., Linden J.F., Paton J.J., Ranson A., Schröder S. (2017). High-Yield Methods for Accurate Two-Alternative Visual Psychophysics in Head-Fixed Mice. Cell Rep..

[bib9] Dana H., Chen T.W., Hu A., Shields B.C., Guo C., Looger L.L., Kim D.S., Svoboda K. (2014). Thy1-GCaMP6 transgenic mice for neuronal population imaging in vivo. PLoS ONE.

[bib10] de G. Matthews A.G., van der Wilk M., Nickson T., Fujii K., Boukouvalas A., León-Villagrá P., Ghahramani Z., Hensman J. (2017). GPflow: A Gaussian Process Library using TensorFlow. J. Mach. Learn. Res..

[bib11] de Lafuente V., Romo R. (2005). Neuronal correlates of subjective sensory experience. Nat. Neurosci..

[bib12] Ding L., Gold J.I. (2010). Caudate encodes multiple computations for perceptual decisions. J. Neurosci..

[bib13] Erlich J.C., Bialek M., Brody C.D. (2011). A cortical substrate for memory-guided orienting in the rat. Neuron.

[bib14] Erlich J.C., Brunton B.W., Duan C.A., Hanks T.D., Brody C.D. (2015). Distinct effects of prefrontal and parietal cortex inactivations on an accumulation of evidence task in the rat. eLife.

[bib15] Fee M.S. (2014). The role of efference copy in striatal learning. Curr. Opin. Neurobiol..

[bib16] Gibbon J. (1977). Scalar expectancy theory and Weber’s law in animal timing. Psychol. Rev..

[bib17] Gilad A., Gallero-Salas Y., Groos D., Helmchen F. (2018). Behavioral Strategy Determines Frontal or Posterior Location of Short-Term Memory in Neocortex. Neuron.

[bib18] Glickfeld L.L., Histed M.H., Maunsell J.H.R. (2013). Mouse primary visual cortex is used to detect both orientation and contrast changes. J. Neurosci..

[bib19] Gold J.I., Shadlen M.N. (2007). The neural basis of decision making. Annu. Rev. Neurosci..

[bib20] Guo Z.V., Li N., Huber D., Ophir E., Gutnisky D., Ting J.T., Feng G., Svoboda K. (2014). Flow of cortical activity underlying a tactile decision in mice. Neuron.

[bib21] Han Y., Kebschull J.M., Campbell R.A.A., Cowan D., Imhof F., Zador A.M., Mrsic-Flogel T.D. (2018). The logic of single-cell projections from visual cortex. Nature.

[bib22] Hanes D.P., Schall J.D. (1996). Neural control of voluntary movement initiation. Science.

[bib23] Hanks T.D., Summerfield C. (2017). Perceptual Decision Making in Rodents, Monkeys, and Humans. Neuron.

[bib24] Hanks T.D., Kopec C.D., Brunton B.W., Duan C.A., Erlich J.C., Brody C.D. (2015). Distinct relationships of parietal and prefrontal cortices to evidence accumulation. Nature.

[bib25] Harvey C.D., Coen P., Tank D.W. (2012). Choice-specific sequences in parietal cortex during a virtual-navigation decision task. Nature.

[bib26] Hensman J., Lawrence N.D., Rattray M. (2013). Hierarchical Bayesian modelling of gene expression time series across irregularly sampled replicates and clusters. BMC Bioinformatics.

[bib27] Hensman J., Matthews A., Ghahramani Z. (2015). Scalable Variational Gaussian Process Classification. arXiv.

[bib28] Hernández A., Nácher V., Luna R., Zainos A., Lemus L., Alvarez M., Vázquez Y., Camarillo L., Romo R. (2010). Decoding a perceptual decision process across cortex. Neuron.

[bib29] Hovde K., Gianatti M., Witter M.P., Whitlock J.R. (2019). Architecture and organization of mouse posterior parietal cortex relative to extrastriate areas. Eur. J. Neurosci..

[bib30] Huk A.C., Shadlen M.N. (2005). Neural activity in macaque parietal cortex reflects temporal integration of visual motion signals during perceptual decision making. J. Neurosci..

[bib31] Jaramillo S., Zador A.M. (2011). The auditory cortex mediates the perceptual effects of acoustic temporal expectation. Nat. Neurosci..

[bib32] Kingma D.P., Ba J. (2014). Adam: A Method for Stochastic Optimization. arXiv.

[bib33] Klein S., Staring M., Murphy K., Viergever M.A.M.A., Pluim J.P.W. (2010). elastix: a toolbox for intensity-based medical image registration. IEEE Trans. Med. Imaging.

[bib34] Kleiner M., Brainard D., Pelli D., Ingling A., Murray R., Broussard C. (2007). What’s new in Psychtoolbox-3?. Perception.

[bib35] Kucukelbir A., Tran D., Ranganath R., Gelman A., Blei D.M. (2017). Automatic Differentiation Variational Inference. J. Mach. Learn. Res..

[bib36] Leinweber M., Zmarz P., Buchmann P., Argast P., Hübener M., Bonhoeffer T., Keller G.B. (2014). Two-photon calcium imaging in mice navigating a virtual reality environment. J. Vis. Exp..

[bib37] Ma Y., Shaik M.A., Kim S.H., Kozberg M.G., Thibodeaux D.N., Zhao H.T., Yu H., Hillman E.M.C. (2016). Wide-field optical mapping of neural activity and brain haemodynamics: considerations and novel approaches. Philos. Trans. R. Soc. Lond. B Biol. Sci..

[bib38] Makino H., Ren C., Liu H., Kim A.N., Kondapaneni N., Liu X., Kuzum D., Komiyama T. (2017). Transformation of Cortex-wide Emergent Properties during Motor Learning. Neuron.

[bib39] Marshel J.H., Garrett M.E., Nauhaus I., Callaway E.M. (2011). Functional specialization of seven mouse visual cortical areas. Neuron.

[bib40] Murakami M., Mainen Z.F. (2015). Preparing and selecting actions with neural populations: toward cortical circuit mechanisms. Curr. Opin. Neurobiol..

[bib41] Murray J.D., Bernacchia A., Freedman D.J., Romo R., Wallis J.D., Cai X., Padoa-Schioppa C., Pasternak T., Seo H., Lee D., Wang X.J. (2014). A hierarchy of intrinsic timescales across primate cortex. Nat. Neurosci..

[bib42] Musall S., Kaufman M.T., Juavinett A.L., Gluf S., Churchland A.K. (2019). Single-trial neural dynamics are dominated by richly varied movements. Nat. Neurosci..

[bib43] Newsome W.T., Britten K.H., Movshon J.A. (1989). Neuronal correlates of a perceptual decision. Nature.

[bib44] Nienborg H., Cumming B.G. (2009). Decision-related activity in sensory neurons reflects more than a neuron’s causal effect. Nature.

[bib45] Odoemene O., Pisupati S., Nguyen H., Churchland A.K. (2018). Visual evidence accumulation guides decision-making in unrestrained mice. J. Neurosci..

[bib46] Okazawa G., Sha L., Purcell B.A., Kiani R. (2018). Psychophysical reverse correlation reflects both sensory and decision-making processes. Nat. Commun..

[bib47] Pachitariu M., Stringer C., Dipoppa M., Schröder S., Rossi L.F., Dalgleish H., Carandini M., Harris K. (2016). Suite2p: beyond 10,000 neurons with standard two-photon microscopy. bioRxiv.

[bib48] Park I.M., Meister M.L.R., Huk A.C., Pillow J.W. (2014). Encoding and decoding in parietal cortex during sensorimotor decision-making. Nat. Neurosci..

[bib49] Pedregosa F., Varoquaux G., Gramfort A., Michel V., Thirion B., Grisel O., Blondel M., Prettenhofer P., Weiss R., Dubourg V. (2011). Scikit-learn: Machine Learning in Python. J. Mach. Learn. Res..

[bib50] Peters A.J., Fabre J.M.J., Steinmetz N.A., Harris K.D., Carandini M. (2021). Striatal activity topographically reflects cortical activity. Nature.

[bib51] Pinto L., Rajan K., DePasquale B., Thiberge S.Y., Tank D.W., Brody C.D. (2019). Task-Dependent Changes in the Large-Scale Dynamics and Necessity of Cortical Regions. Neuron.

[bib52] Pinto L., Tank D.W., Brody C.D. (2020). Multiple timescales of sensory-evidence accumulation across the dorsal cortex. bioRxiv.

[bib53] Pologruto T.A., Sabatini B.L., Svoboda K. (2003). ScanImage: flexible software for operating laser scanning microscopes. Biomed. Eng. Online.

[bib54] Poort J., Khan A.G., Pachitariu M., Nemri A., Orsolic I., Krupic J., Bauza M., Sahani M., Keller G.B., Mrsic-Flogel T.D., Hofer S.B. (2015). Learning Enhances Sensory and Multiple Non-sensory Representations in Primary Visual Cortex. Neuron.

[bib55] Raposo D., Kaufman M.T., Churchland A.K. (2014). A category-free neural population supports evolving demands during decision-making. Nat. Neurosci..

[bib56] Rasmussen C.E. (2006). Gaussian processes for machine learning.

[bib57] Ratzlaff E.H., Grinvald A. (1991). A tandem-lens epifluorescence macroscope: hundred-fold brightness advantage for wide-field imaging. J. Neurosci. Methods.

[bib58] Roitman J.D., Shadlen M.N. (2002). Response of neurons in the lateral intraparietal area during a combined visual discrimination reaction time task. J. Neurosci..

[bib59] Romo R., Hernández A., Zainos A., Lemus L., Brody C.D. (2002). Neuronal correlates of decision-making in secondary somatosensory cortex. Nat. Neurosci..

[bib60] Ruediger S., Scanziani M. (2020). Learning speed and detection sensitivity controlled by distinct cortico-fugal neurons in visual cortex. eLife.

[bib61] Scott B.B., Constantinople C.M., Akrami A., Hanks T.D., Brody C.D., Tank D.W. (2017). Fronto-parietal Cortical Circuits Encode Accumulated Evidence with a Diversity of Timescales. Neuron.

[bib62] Shadlen M.N., Newsome W.T. (1996). Motion perception: seeing and deciding. Proc. Natl. Acad. Sci. USA.

[bib63] Shuler M.G., Bear M.F. (2006). Reward timing in the primary visual cortex. Science.

[bib64] Siegel M., Buschman T.J., Miller E.K. (2015). Cortical information flow during flexible sensorimotor decisions. Science.

[bib65] Silasi G., Xiao D., Vanni M.P., Chen A.C.N., Murphy T.H. (2016). Intact skull chronic windows for mesoscopic wide-field imaging in awake mice. J. Neurosci. Methods.

[bib66] Snelson E., Ghahramani Z. (2006). Variable noise and dimensionality reduction for sparse gaussian processes. arXiv.

[bib67] Snelson E., Ghahramani Z., Rasmussen C.E., Thrun S., Saul L.K., Schölkopf B. (2004). Warped Gaussian Processes. Advances in Neural Information Processing Systems.

[bib68] Steinmetz N.A., Zatka-Haas P., Carandini M., Harris K.D. (2019). Distributed coding of choice, action and engagement across the mouse brain. Nature.

[bib69] Stringer C., Pachitariu M., Steinmetz N., Reddy C.B., Carandini M., Harris K.D. (2019). Spontaneous behaviors drive multidimensional, brainwide activity. Science.

[bib70] Summerfield C., de Lange F.P. (2014). Expectation in perceptual decision making: neural and computational mechanisms. Nat. Rev. Neurosci..

[bib71] Vivarelli F., Williams C.K.I., Kearns M.J., Solla S.A., Cohn D.A. (1999). Discovering Hidden Features with Gaussian Processes Regression. Advances in Neural Information Processing Systems.

[bib72] Wekselblatt J.B., Flister E.D., Piscopo D.M., Niell C.M. (2016). Large-scale imaging of cortical dynamics during sensory perception and behavior. J. Neurophysiol..

[bib73] Zatka-Haas P., Steinmetz N.A., Carandini M., Harris K.D. (2018). Distinct contributions of mouse cortical areas to visual discrimination. bioRxiv.

[bib74] Znamenskiy P., Zador A.M. (2013). Corticostriatal neurons in auditory cortex drive decisions during auditory discrimination. Nature.

