# Supplementary material for: Mesoscale cortical dynamics reflect the interaction of sensory evidence and temporal expectation during perceptual decision-making
Source: Neuron. 2021 Jun 2;109(11):1861–1875.e10. doi: 10.1016/j.neuron.2021.03.031 (PMC8186564; doi:10.1016/j.neuron.2021.03.031)
Supplement: Document S1. Figures S1–S11 [file mmc1.pdf]

Neuron, Volume *109*

Supplemental information

Mesoscale cortical dynamics reflect the interaction of sensory evidence and temporal expectation during perceptual decision-making

Ivana Orsolic, Maxime Rio, Thomas D. Mrsic-Flogel, and Petr Znamenskiy

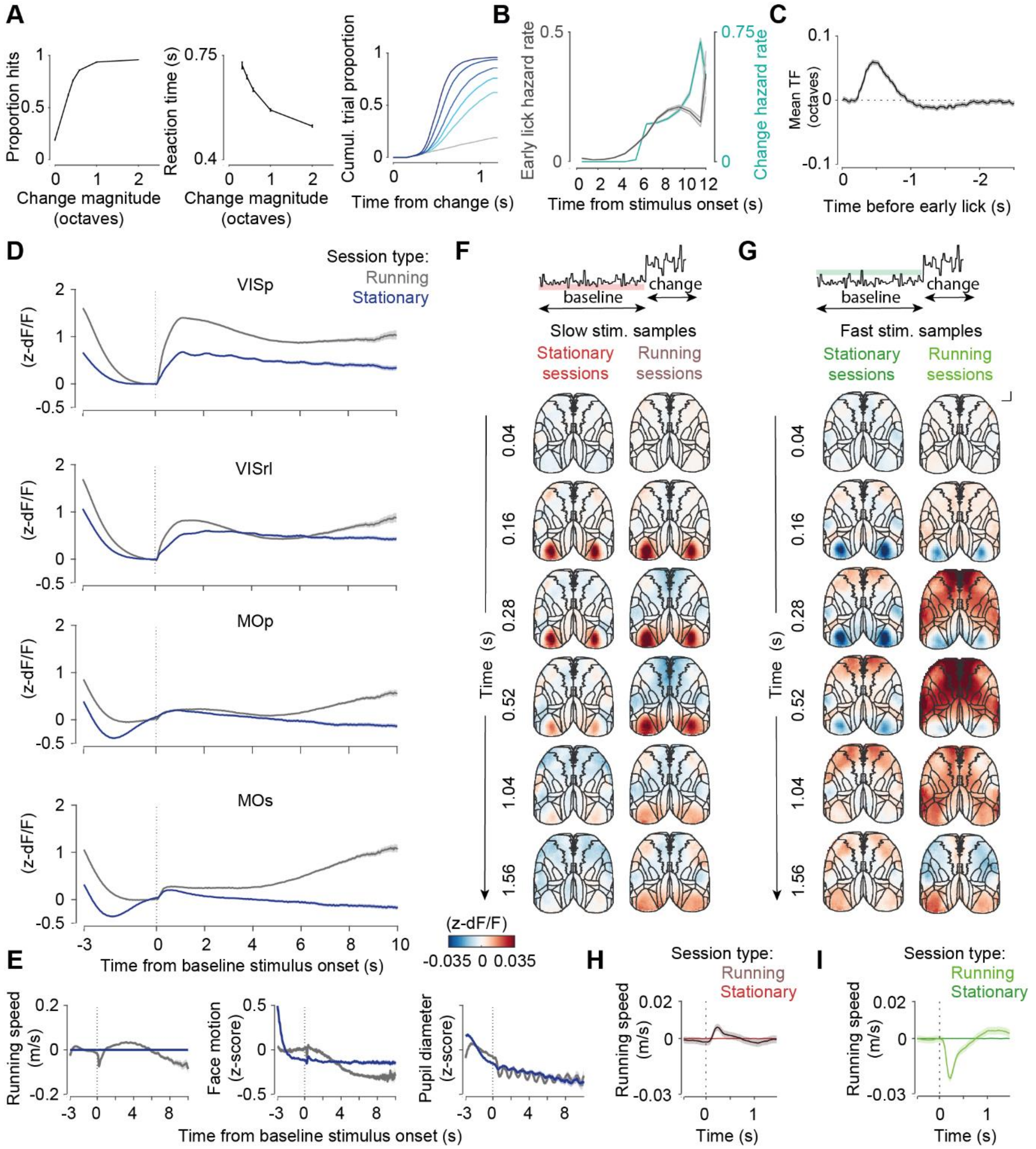

Figure S1. Mice modulate their running behavior during the baseline stimulus period as a function of both time and stimulus. Related to Figure 1.

A. Performance of animals in the running version of the task. Detection rate and median reaction times are modulated by change magnitude (6 mice, error bars are 95% CI). Right - cumulative distribution of reaction times across stimulus changes. Colors follow same convention as in Figure 1.

B. Early lick hazard rate (gray) and change hazard rate (cyan).

C. Average stimulus TF preceding licks during the baseline stimulus (6 mice, shading is 95% CI).

D-E. Mean z-scored responses of selected cortical areas around the onset of baseline stimulus in stationary (blue) and running (gray) version of the task (D), quantification of overt

movement (E). Traces are corrected for onset responses (values at time 0 for running speed: stationary: 8.35×10^{-5}, running: 0.45 m/s; face motion: stationary: -0.49, running: 0.01; pupil diameter: stationary: -0.11, running: 0.34).

F-G. Maps of mean z-scored fluorescence responses to anti-licking (slow samples, F) and pro-licking (fast samples, G) subthreshold stimulus fluctuations in stationary (left, fast samples: N = 41194 frames, slow samples: 42253 frames, 6894 trials, 47 sessions, 6 mice) and running mice (right, fast samples: N = 42004 frames, slow samples: 42870 frames, reference samples: 481756, 5930 trials, 37 sessions, 6 mice). Scale bar – 1 mm.

H. Slow samples in running but not stationary mice are followed by an increase in average running speed.

I. Fast samples in running but not stationary mice are followed by a reduction in average running speed.

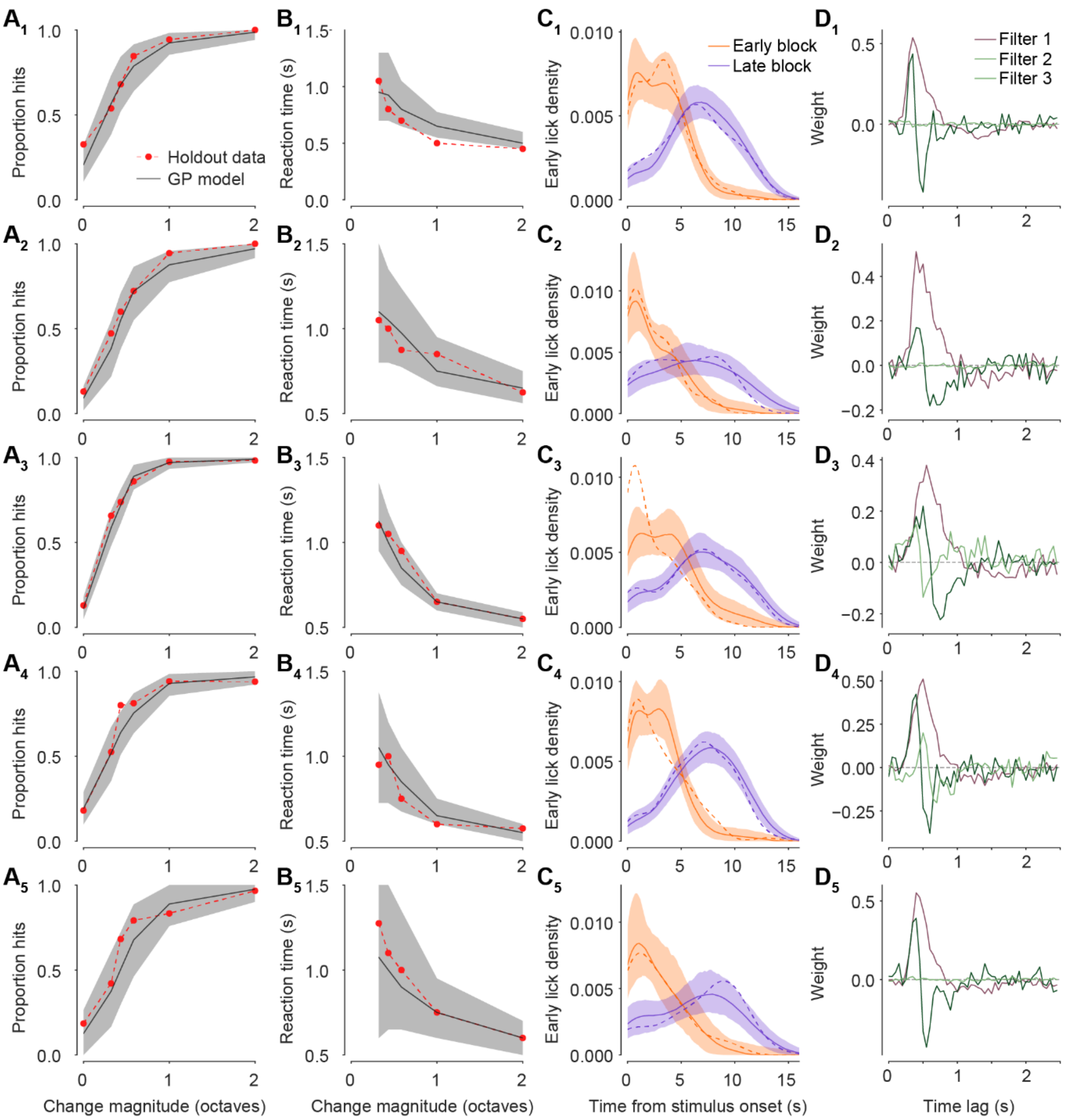

Figure S2. GP classification model captures mouse behavior and identifies similar stimulus filters across mice. Related to Figure 2.

Model predictions and holdout data for 5 additional mice; notation as in Figure 2C-F. Columns show psychometric curves (**A**$_{1\text{-}5}$), chronometric curves (**B**$_{1\text{-}5}$), early lick timing distributions (**C**$_{1\text{--}5}$) and the top three stimulus history filters (**D**$_{1\text{-}5}$).

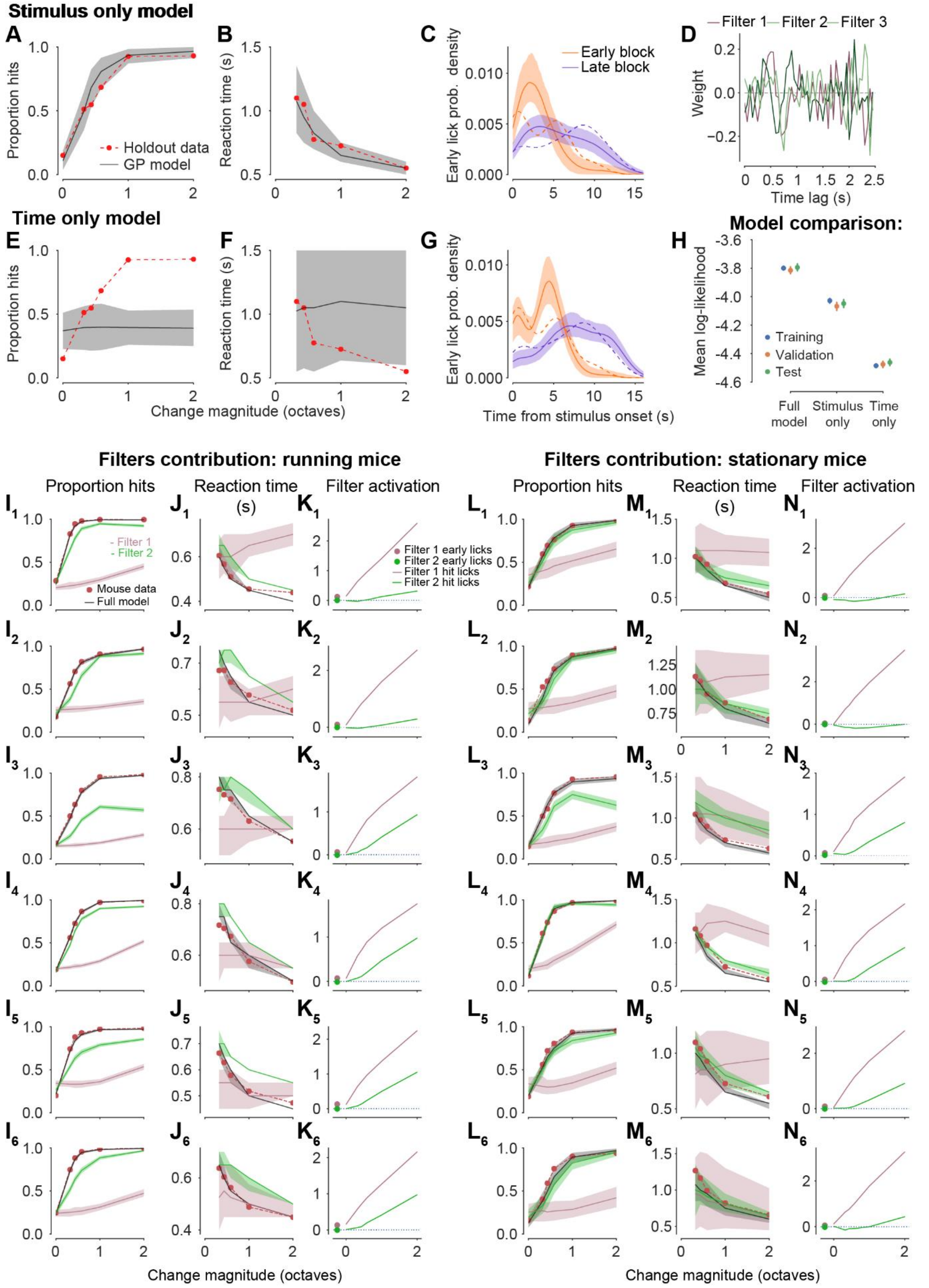

Figure S3. Contributions of time, stimulus and stimulus filters to model performance. Related to Figure 2.

A-D. A model that only receives stimulus information captures psychometric (A) and chronometric (B) performance but does not fully account for the timing of early licks (C) or identify both stimulus filters (D). Notation as in Figure 2C-F.
E-G. A model that only receives timing information does not account for animal's psychometric (E) and chronometric (F) performance.
H. Models including both stimulus and time information outperform those based on the stimulus or time alone, as quantified by mean log-likelihood including data from all 6 mice in Figures 2, S2. Error bars – 95% confidence interval, estimated by resampling trials.
I-N. Contribution of the top two stimulus filters to model performance. Psychometric ($I_{1\text{-}6}$, $L_{1\text{-}6}$) and chronometric ($J_{1\text{-}6}$, $M_{1\text{-}6}$) curves for the mouse data, full model, and ablated models with filter 1 or filter 2 coefficients set to 0, and filter activations at the time of mouse licks ($K_{1\text{-}6}$, $N_{1\text{--}6}$). **I-K** – model performance for running sessions without hazard rate manipulations; **L-N** – model performance for stationary sessions with hazard rate manipulations. Since this analysis did not aim to evaluate the prediction performance of the model but instead to compare the performance of the full and ablated models, this figure includes the entire dataset (including training, validation, and test data).

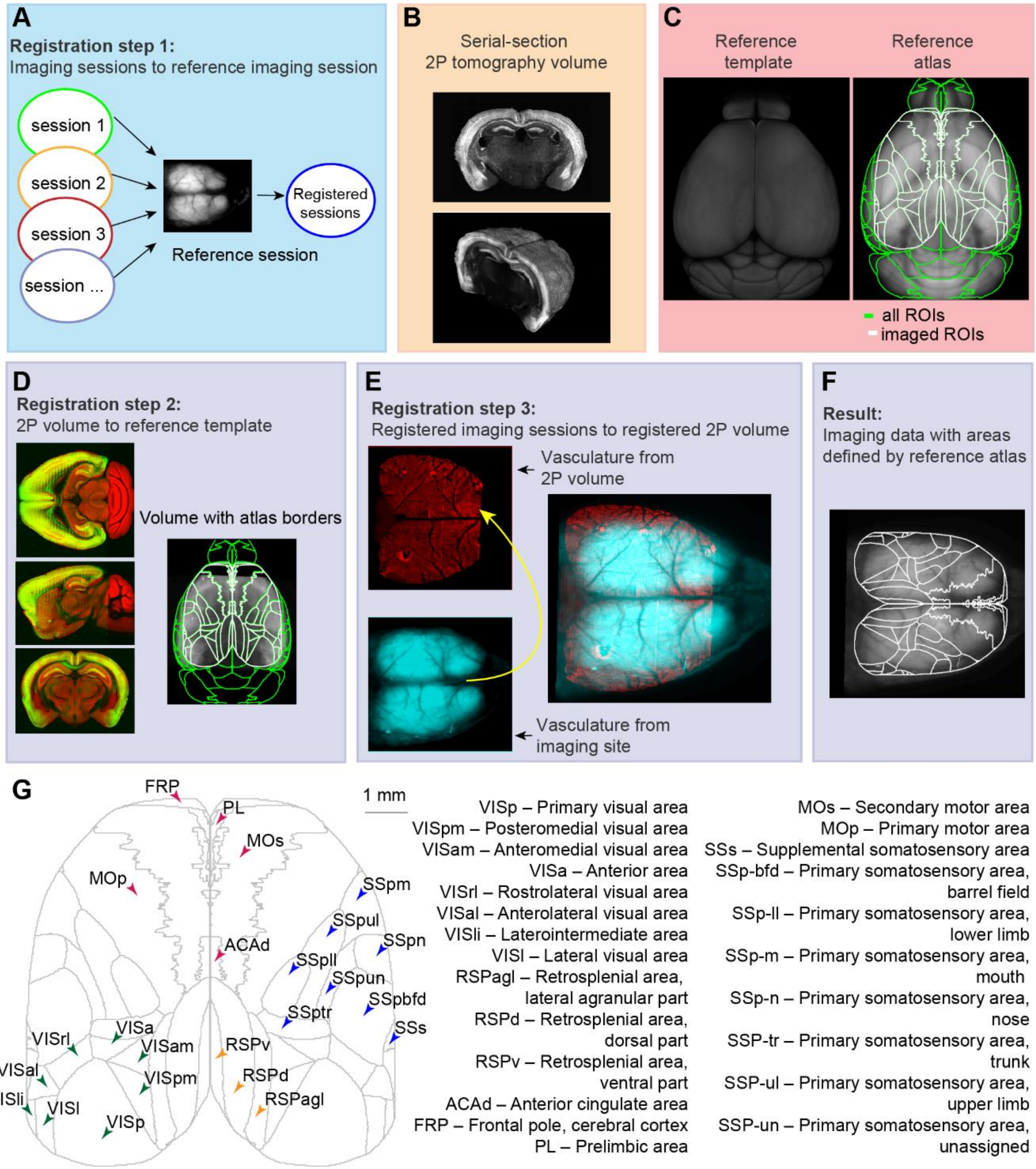

Figure S4. Registration of wide-field imaging data to the reference atlas. Related to Figure 3.

A. All imaging sessions were registered to a single reference imaging session for each brain using the vasculature pattern.

B. After the *in vivo* imaging experiments, brains were serially sectioned and imaged using a two-photon microscope. Imaged frames were tiled to reconstruct sections (top), and sections were reconstructed to 3D volumes (bottom).

C. Reference brain template dorsal view (left) and projection of reference atlas (right) were obtained from the Allen Institute for Brain Science. Borders of all dorsal areas are shown in green. In white are borders trimmed to the extent of the imaging site.

D. The sample volume from **B** (in green) was registered to reference template from **C** (in red). Right - atlas projection overlay over projection of registered two-photon volume.

E. Vasculature pattern of the reference imaging session (**A**) was registered to the vasculature pattern of the registered two-photon volume from **D**.
F. The resulting transformation aligned wide-field imaging data to the reference atlas coordinate frame.
G. Area boundaries and abbreviations used.

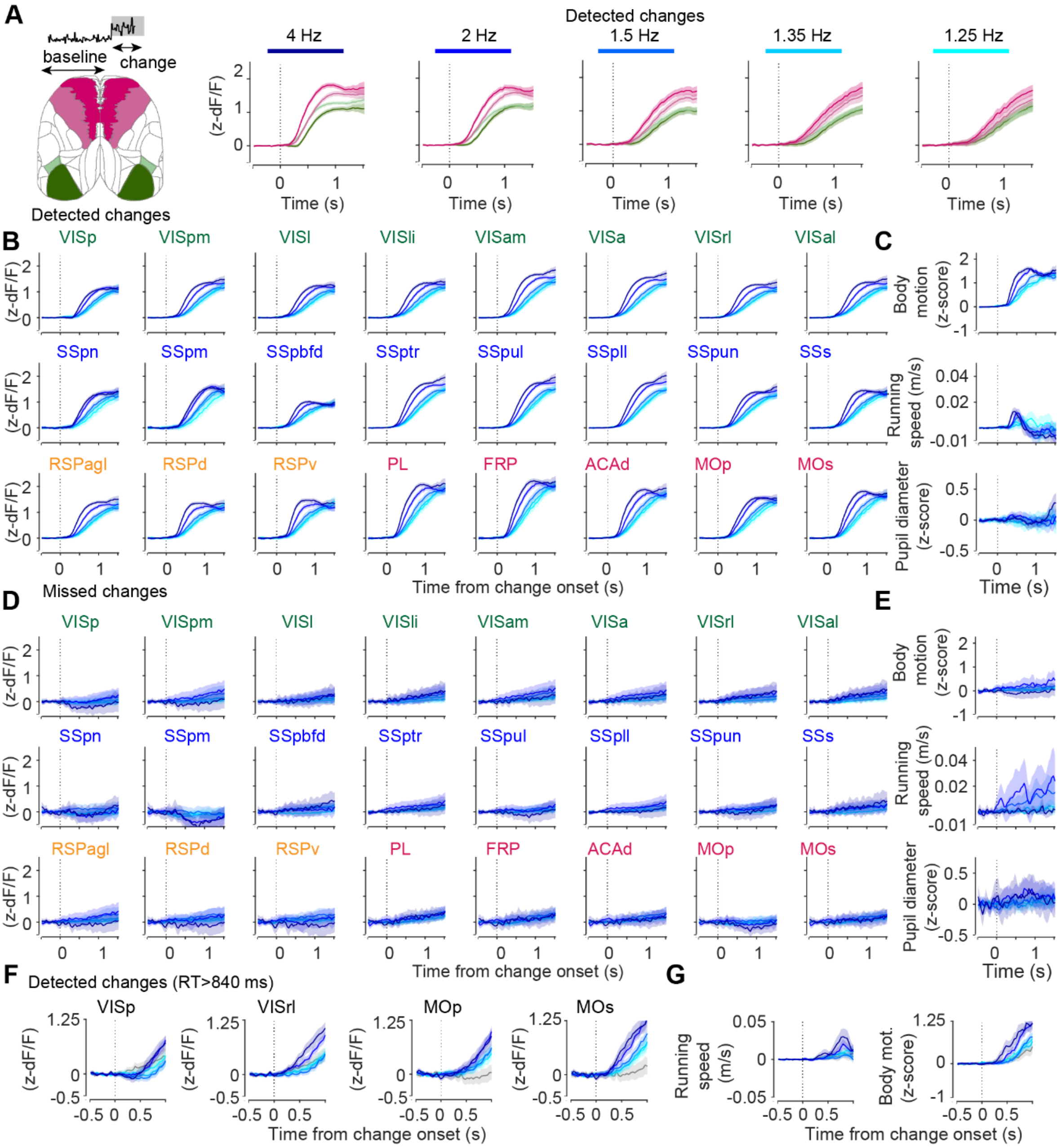

Figure S5. Wide-field calcium responses during the change period across to all imaged cortical areas. Related to Figure 4.

A. Time courses of responses in VISp, VISrl, MOp, and MOs aligned to change onset on hit trials across the change strengths.

B-C. Mean z-scored fluorescence of all imaged cortical areas (B) and quantification of overt movements (C) aligned to change onset on hit trials.

D-E. Mean z-scored fluorescence of all imaged cortical areas (D) and quantification of overt movements (E) aligned to change onset on miss trials.

F. Mean z-scored fluorescence of selected cortical areas and quantification of overt movements across change strengths aligned to change onsets on hit trials where lick happened at least 0.84 s after the change point.

G. Body movements preceding licks accompany increase in activity on hit trials (F).

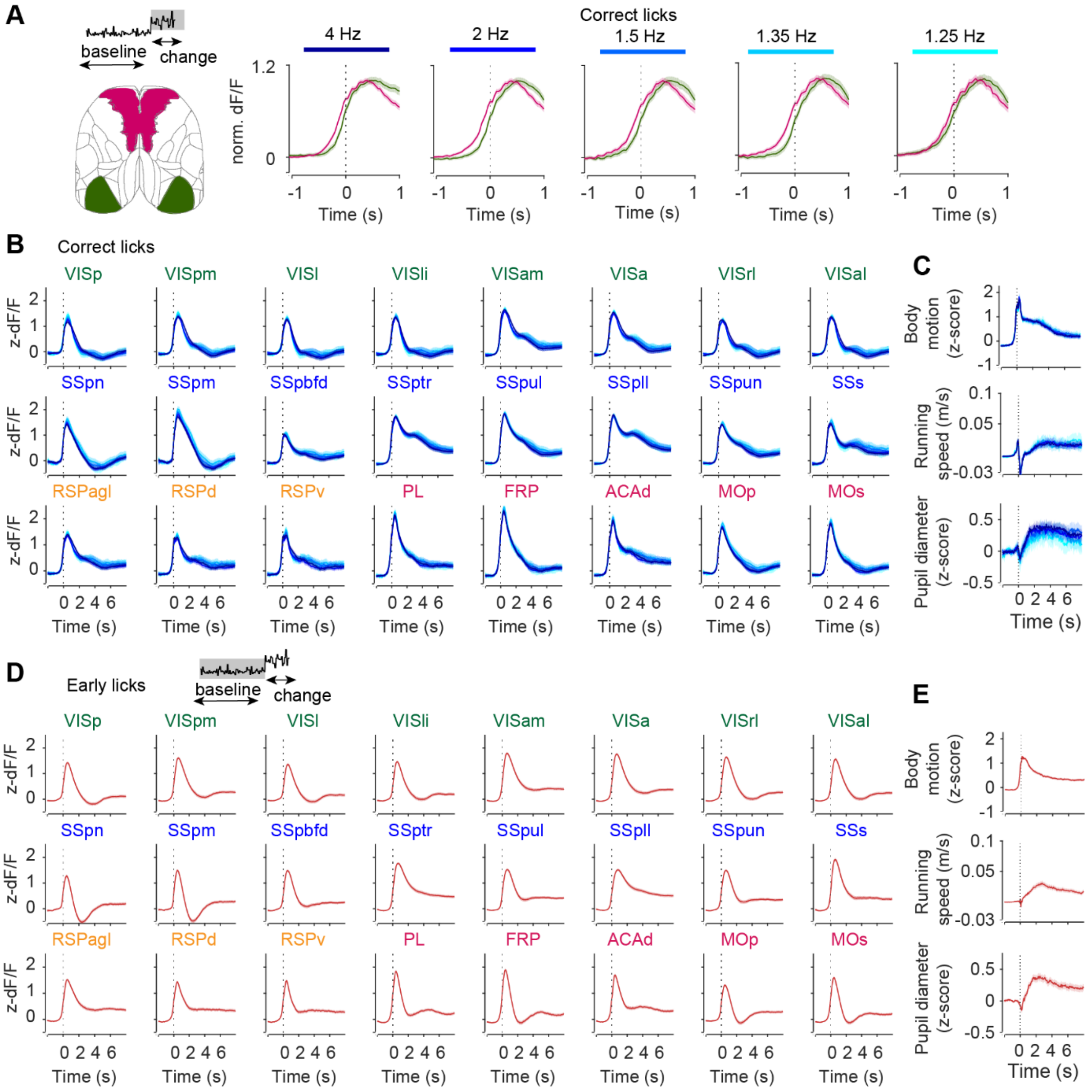

Figure S6. Wide-field calcium responses aligned to licks across to all imaged cortical areas. Related to Figure 4.

A. Normalized time courses of responses in VISp and MOs aligned to detection of correct licks across the change strengths.

B-C. Mean z-scored fluorescence of all imaged cortical areas (A) and quantification of overt movements (B) aligned to licks on hit trials.

D-E. Mean z-scored fluorescence of all imaged cortical areas (C) and quantification of overt movements (D) aligned to early licks (licks during the baseline period).

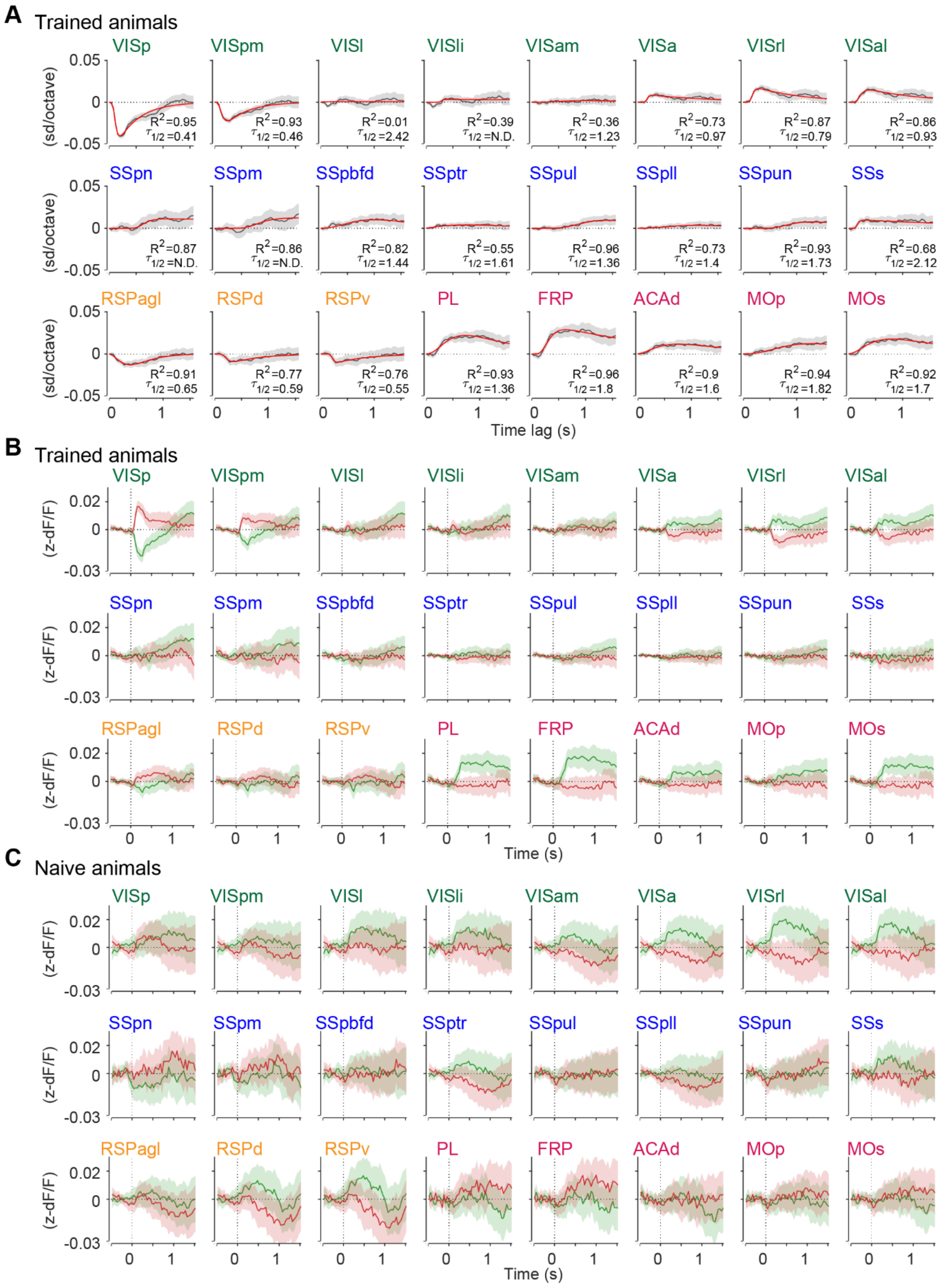

Figure S7. Responses to baseline stimulus fluctuations across all imaged areas. Related to Figure 5.

A. Time course of regression coefficients of wide-field fluorescence against baseline stimulus TF in all imaged cortical areas; regression coefficients (gray, 95% CI) and multiexponential fits (red).

B-C. Mean z-scored fluorescence of all imaged cortical areas aligned to anti-licking (slow stimulus samples, red) and pro-licking (fast stimulus samples, green) baseline stimulus fluctuations in (B) trained (fast stimulus samples: N = 41194 frames, slow stimulus samples: 42253 frames, 6894 trials, 47 sessions, 6 mice) and (C) naive mice (fast stimulus samples: N = 14462 frames, slow stimulus samples: 14674 frames, 1680 trials, 10 sessions, 3 mice). Shading is 95% CI.

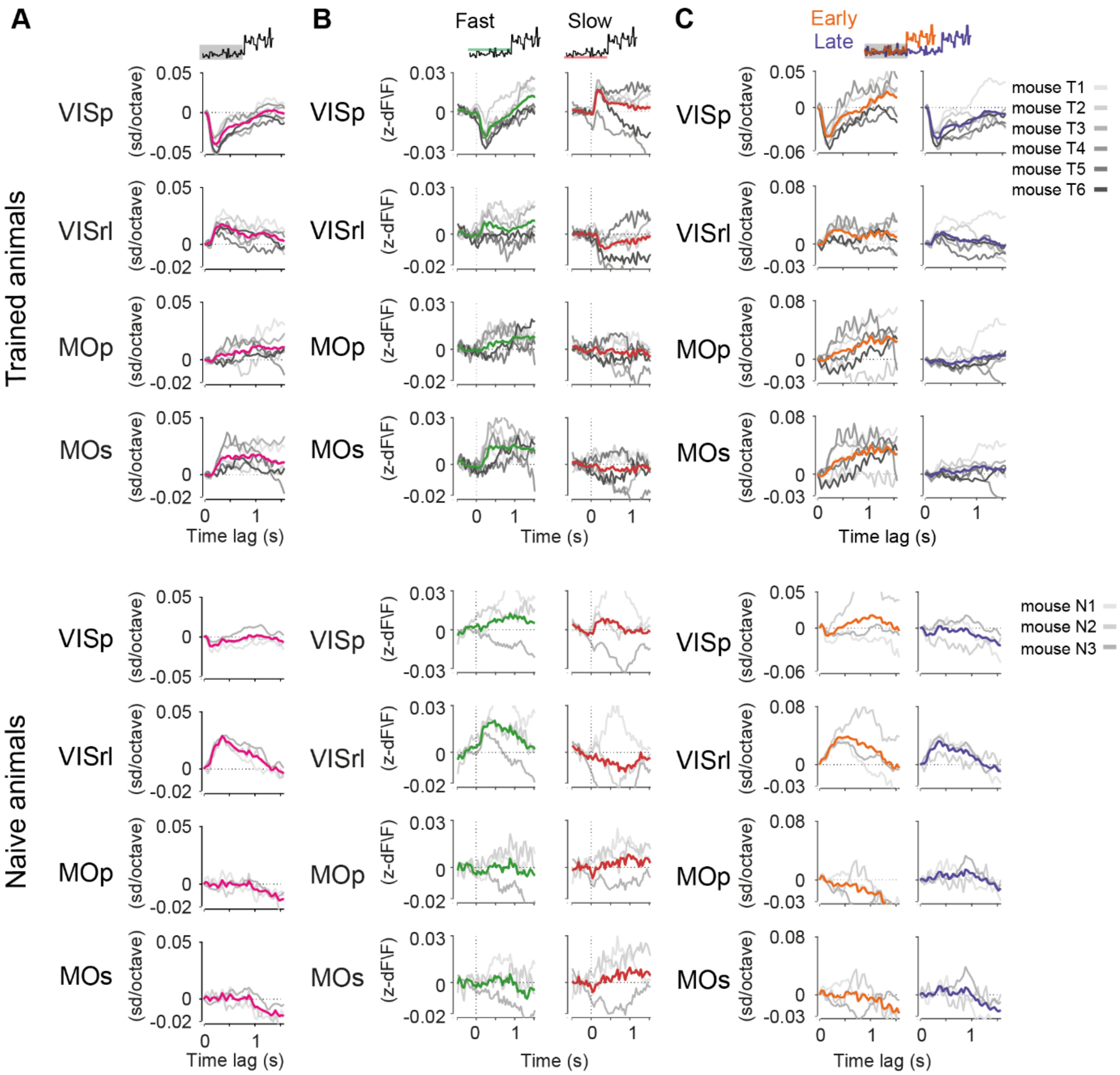

Figure S8. Wide-field responses to baseline stimulus fluctuations in individual mice. Related to Figure 5.

A. Regression coefficients of wide-field fluorescence against baseline stimulus TF in trained (top) and naïve (bottom) animals across time lags, related to Figure 5B. In all panels, colored traces show analyses of activity pooled across all mice.

B. Mean z-scored fluorescence responses of selected cortical areas in trained (top) and naïve (bottom) animals following fast (left) or slow (right) stimulus samples, related to Figure 5G.

C. Regression coefficients of wide-field fluorescence against baseline stimulus temporal frequency during 0-6 s of the trial in early (left) and late (right) change blocks in trained (top) and naïve (bottom) animals, related to Figure 7A.

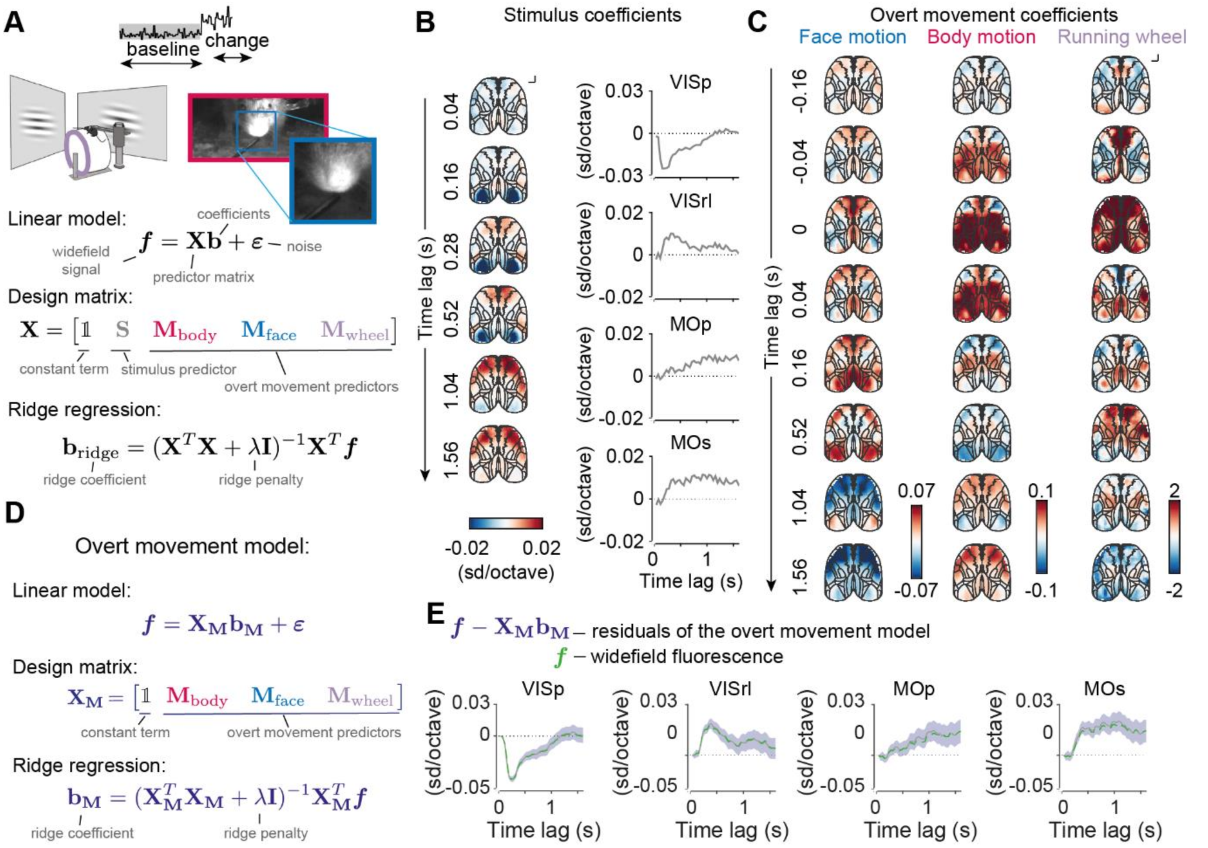

Figure S9. Cortical activation during baseline stimulus fluctuations is not explained by small overt movements. Related to Figure 5.

A. To account for small movements captured by running wheel or face camera (top) during the baseline fluctuations of the stimulus we fit the wide-field fluorescence using a liner model that included stimulus and overt movement regressors using ridge regression (bottom).

B. Regression coefficients corresponding to stimulus fluctuations during the baseline period show activation profile consistent with the previous analysis (Figure 5A).

C. Maps of regression coefficients corresponding to overt body movements during the baseline period.

D. A linear model of wide-field fluorescence only including overt movement predictors.

E. Time courses of responses of cortical areas to fluctuations in the baseline stimulus (as in Figure 5B) after accounting for overt movements following regression model in D (purple) compared to uncorrected wide-field fluorescence (green).

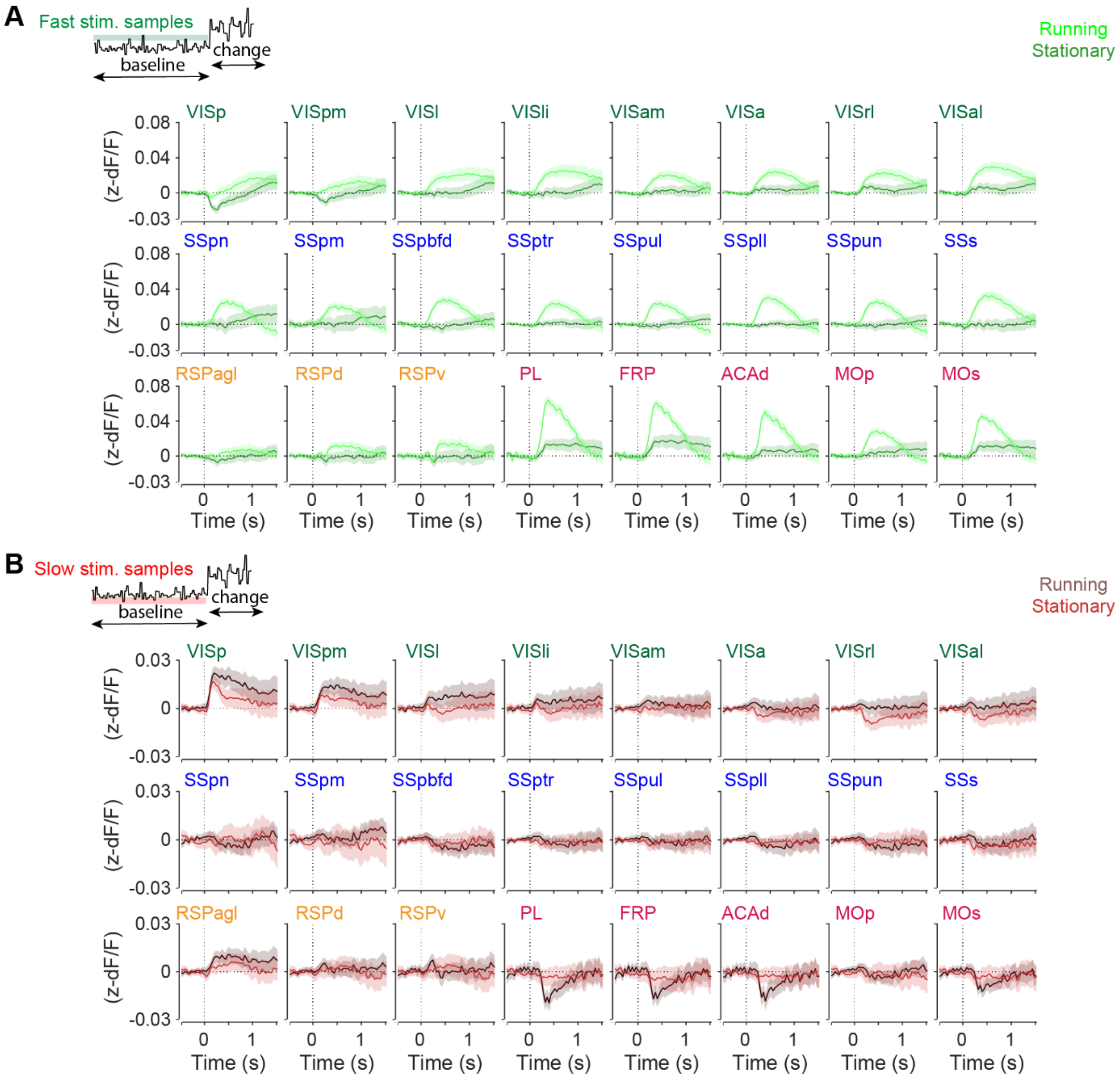

Figure S10. Comparison of responses to fast and slow stimulus samples during running and stationary sessions across all imaged cortical areas. Related to Figure 5.

A. Mean z-scored fluorescence of all imaged cortical areas aligned to pro-licking (fast) subthreshold stimulus fluctuations during sessions when mice were required to remain stationary (dark green) or free to run on the wheel (light green).

B. Mean z-scored fluorescence of all imaged cortical areas aligned to anti-licking (slow) subthreshold stimulus fluctuations during sessions when mice were required to remain stationary (red) or free to run on the wheel (black).

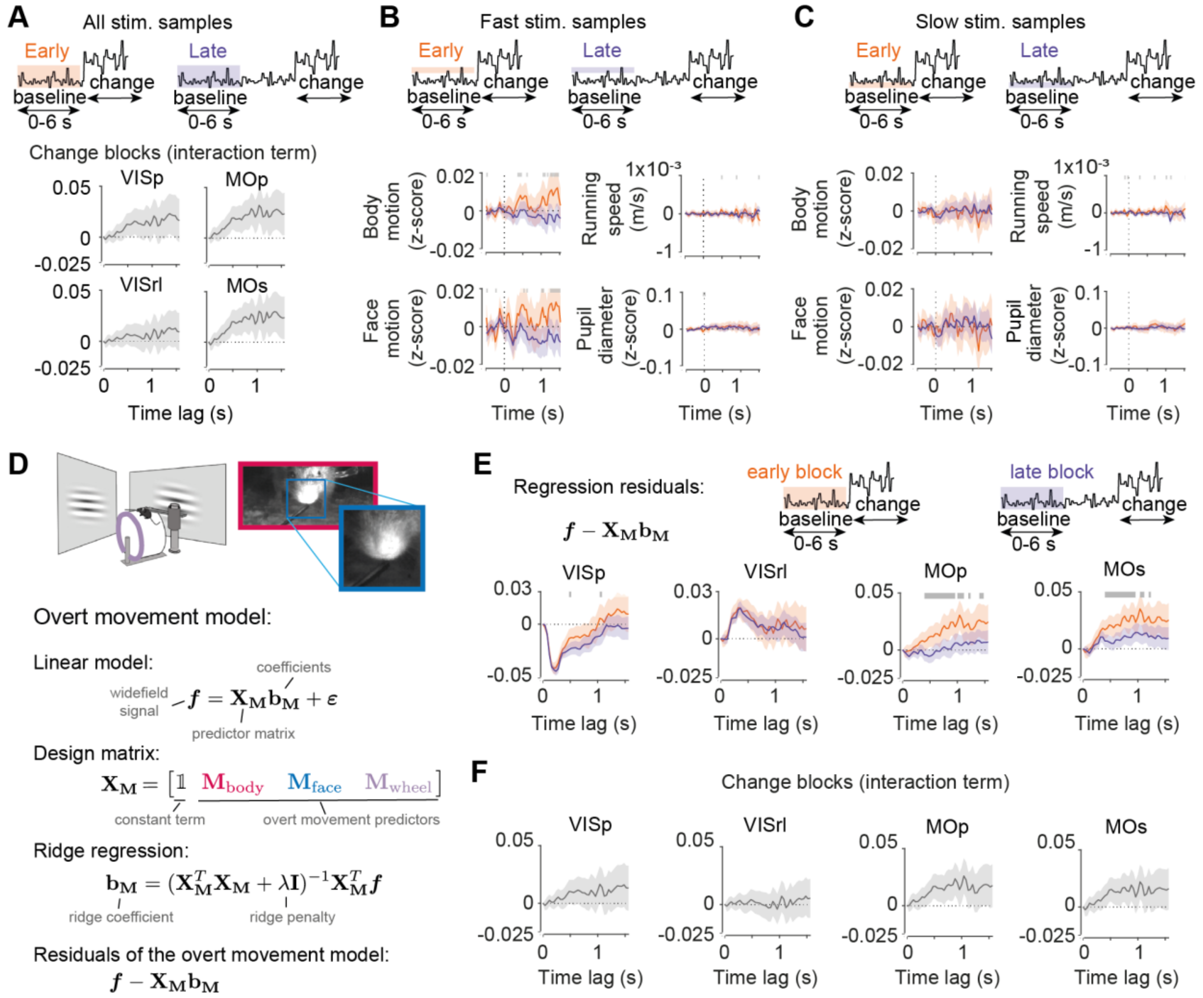

Figure S11. Differences in small overt movements do not explain modulation of motor cortex responses by temporal expectation during the baseline period. Related to Figure 7.

A. Interaction term capturing the effect of temporal expectation on the relationship between fluorescence and baseline stimulus fluctuations. Gray bars in Figure 7A indicate periods when the interaction is significantly different from 0 ($p<0.05$, see Methods).

B-C. Quantification of overt movements in early and late change blocks following fast (B) and slow (C) stimulus samples (shading is 95% CI; gray bars indicate periods where the two traces are significantly different (two sample t-test, $p<0.05$).

D. To account for small movements during the baseline fluctuations of the stimulus we fit a linear model of wide-field fluorescence that included overt movement as predictors using ridge regression.

E. We split the residuals of the fit in (E) into early and late expectation block and repeated the regression analysis as in Figure 7A. Gray bars indicate where when the effect of temporal expectation is significantly different from 0 ($p<0.05$).

F. Interaction term capturing the effect temporal expectation on the relationship between residuals of the fit in (E) and baseline stimulus fluctuations. Highlighted in panel (E) are periods when the interaction is significantly different from 0 ($p<0.05$).
